# The Exposed Proteomes of *Brachyspira hyodysenteriae* and *B. pilosicoli*

**DOI:** 10.3389/fmicb.2016.01103

**Published:** 2016-07-21

**Authors:** Vanessa Casas, Santiago Vadillo, Carlos San Juan, Montserrat Carrascal, Joaquin Abian

**Affiliations:** ^1^Consejo Superior de Investigaciones Científicas/UAB Proteomics Laboratory, Instituto de Investigaciones Biomedicas de Barcelona–Consejo Superior de Investigaciones Científicas, Institut d'investigacions Biomèdiques August Pi i SunyerBarcelona, Spain; ^2^Departamento Sanidad Animal, Facultad de Veterinaria, Universidad de ExtremaduraCáceres, Spain

**Keywords:** shotgun proteomics, *Brachyspira*, surface proteins, virulence factors, membrane shaving

## Abstract

*Brachyspira hyodysenteriae* and *Brachyspira pilosicoli* are well-known intestinal pathogens in pigs. *B. hyodysenteriae* is the causative agent of swine dysentery, a disease with an important impact on pig production while *B. pilosicoli* is responsible of a milder diarrheal disease in these animals, porcine intestinal spirochetosis. Recent sequencing projects have provided information for the genome of these species facilitating the search of vaccine candidates using reverse vaccinology approaches. However, practically no experimental evidence exists of the actual gene products being expressed and of those proteins exposed on the cell surface or released to the cell media. Using a cell-shaving strategy and a shotgun proteomic approach we carried out a large-scale characterization of the exposed proteins on the bacterial surface in these species as well as of peptides and proteins in the extracellular medium. The study included three strains of *B. hyodysenteriae* and two strains of *B. pilosicoli* and involved 148 LC-MS/MS runs on a high resolution Orbitrap instrument. Overall, we provided evidence for more than 29,000 different peptides pointing to 1625 and 1338 different proteins in *B. hyodysenteriae* and *B. pilosicoli*, respectively. Many of the most abundant proteins detected corresponded to described virulence factors and vaccine candidates. The level of expression of these proteins, however, was different among species and strains, stressing the value of determining actual gene product levels as a complement of genomic-based approaches for vaccine design.

## Introduction

The genus *Brachyspira* (previously *Treponema, Serpula*, and *Serpulina*) includes several pathogenic species affecting humans and other animals such as pigs, dogs, and birds. In pigs, *Brachyspira hyodysenteriae* and *Brachyspira pilosicoli* are well-known intestinal pathogens. These species are flagellated, anaerobic, aerotolerant Gram-negative spirochetes that inhabit the large intestine, where they are intimately associated with the colonic mucosa. *B. hyodysenteriae*, an obligate anaerobe with strong β-hemolysis on blood agar, is the causative agent of swine dysentery (Taylor and Alexander, 1971; Harris et al., 1972). *B. hyodysenteriae* colonizes the large intestine and can be found on the luminal surface and within the crypts of the caecum, colon, and rectum. The first evidence of disease is usually soft, yellow to gray feces that usually progress to mucohemorrhagic diarrhea. On the other hand, *B. pilosicoli* (weakly β-hemolytic) produces porcine intestinal/colonic spirochetosis, with gray-wet diarrhea, sometimes with mucus, and occasionally mucohemorrhagic (Mappley et al., 2012).

Swine dysentery, with a mortality rate of 50–90% (Alvarez-Ordóñez et al., 2013), is a disease with an important impact on pig production due to the costs associated with mortality, morbidity, inefficient production, and continual in-feed medication of the animals. Although the disease can affect animals of all ages, it is rarely detected in piglets younger than 3 weeks of age; it occurs more frequently during growing/finishing periods, thereby aggravating economic losses.

Strategies to treat these diseases include the use of antibiotics such as tiamulin, valnemulin, tylosin, tylvalosin, and lincomycin. Unfortunately, the emergence of *B. hyodysenteriae* strains that are resistant to one or several of these antibiotics has been reported in several countries in Europe and Asia and in the US (Alvarez-Ordóñez et al., 2013; Rugna et al., 2015). Although it has long been known that pigs generate resistance to *B. hyodysenteriae* (Joens et al., 1979) after recovering from an infection, no vaccine is currently available. Administration of killed or attenuated bacteria has been of limited success (Alvarez-Ordóñez et al., 2013). Several bacterial recombinant proteins, including membrane and flagellar proteins, have been tested as candidates for this purpose. Experimental infection with the outer-membrane lipoprotein Bhlp29.7 of *B. hyodysenteriae* (also known as BmpB or Blpa) resulted in a 50% reduction in the incidence of disease (La et al., 2004). The search for possible vaccine candidates has been facilitated by the publication of the genome sequences of *B. hyodysenteriae* (WA1 strain; Bellgard et al., 2009) and *B. pilosicoli* (Wanchanthuek et al., 2010). This allows *in silico* analysis of the full genome sequence in the search of possible vaccine candidates that can be expressed and screened. Song et al. demonstrated the potential of this reverse vaccinology approach in a study in which partial genomic data from *B. hyodysenteriae* were used to identify 19 ORF-encoding candidate proteins, including lipoproteins, proteases, toxins, flagella-associated proteins, and membrane proteins. Although the results were not conclusive, a prototype vaccine prepared from four of the recombinant proteins produced antibodies in pigs, and conferred some protection against infection (Song et al., 2009). More recently, a US patent was registered for the development of a vaccine that is proposed to include up to 33 bacterial gene candidates selected from outer-surface and secreted proteins and from virulence factors described in public databases (Bellgard et al., 2015).

The characterization of the secreted and surface-exposed proteins of *B. hyodysenteriae* and *B. pilosicoli* is thus of special interest both for the development of vaccines and for the identification of factors involved in *Brachyspira* infection. Due to their localization, these protein groups are key for the induction of the host immune response (Zagursky and Russell, 2001; Grandi, 2010). As for other pathogens, proteins exposed on the surface of *B. hyodysenteriae* and *B. pilosicoli* play an important role in colonization and disease expression (Trott et al., 2001; Gömmel et al., 2013). On the other hand, secreted proteins such as β-hemolysin, which is considered a major virulence factor in *B. hyodysenteriae*, can act as cytotoxins against the host (Barth et al., 2012).

In reverse vaccinology approaches, vaccine candidates are searched in the microbial genome, mainly among predicted secreted and outer-membrane proteins and lipoproteins. Protein location predictions based on homology comparisons as well as on predictions of the actual levels of the protein molecules in that location have, however, an inherent degree of uncertainty. For example, most secreted proteins are synthesized as precursors with N-terminal signal sequences, but a significant fraction of secreted proteins are secreted by non-classical pathways that do not involve signal peptides (Armengaud et al., 2012). Although signal peptides are necessary for the targeting of many proteins to the membrane-embedded export machinery, the presence of an N-terminal signal peptide does not necessarily mean that the protein will be secreted; it could be released in the periplasmic space, or anchored to the outer membrane. Another important point to be considered is that some secreted proteins are not free in the extracellular milieu; instead, they remain attached to outer membrane components or to macromolecular structures such as flagella. Moreover, application of bacterial prediction algorithms to spirochetes can give inaccurate results due to the high plasticity of the lipobox in these bacteria compared to that of other Gram-negative species (Setubal, 2006). In this context and in comparison with *in silico* approaches, direct analysis of the bacterial proteome using proteomics approaches can provide a more accurate description of the protein profile in a given subcellular location.

Proteomics analysis of the cell surface can give a high-resolution view of the molecular components exposed by the cell, the surfaceome. The surfaceome includes membrane integral proteins as well as other proteins, such as secreted or exported proteins, that are bound to the outer membrane. One efficient method of characterizing the protein sequences exposed to the cellular milieu is cell “shaving.” This strategy uses proteases to partially digest intact cells, resulting in preferential cleavage of the exposed portions of proteins. The resulting peptides are released to the supernatant and can then be identified by mass spectrometry (Solis et al., 2010). This method has been previously used with Gram-negative (Gesslbauer et al., 2012) and Gram-positive bacteria (Tjalsma et al., 2008). One surface-associated protein identified using this strategy has been validated in mice as a potential vaccine candidate (Doro et al., 2009).

On the other hand, direct analysis of the cell milieu provides a view of the extracellular proteome or exoproteome. As defined by Desvaux et al. (2009), the exoproteome includes actively secreted proteins as well as other extracellular, non-secreted proteins resulting from cell lysis, cell friction, and protein degradation, which can be also relevant to immune recognition and pathogenesis. Relevant exoproteome components can range from full-size, high-mass proteins to smaller protein fragments, and oligopeptides. Due to the potential relevance of extracellular oligopeptides, e.g., as protease inhibitors or to cell communication, specific characterization of these components, grouped under the term “exopeptidome,” would be desirable. Bottom-up shotgun proteomics involves, however, technical limits to the characterization of protein fragments and to differentiation between such fragments and full-length proteins. Conventional shotgun proteomics approaches to protein identification based on sequence analysis of proteome digests identify a protein on the basis of the characterization of several of its peptides. Although analysis of protein coverage can provide hints as to whether a given set of peptides reflects the presence of a complete protein, a protein fragment, or a polypeptide, a confident assignation is generally not possible.

Despite the fact that many genome sequences are already available, thus allowing protein characterization in databases, proteomic information for *Brachyspira* species is still scarce. Only a few outer membrane proteins of the genus have been characterized to date. Only 14 proteins are described in Uniprot with existence evidenced at the protein level, and only four of these are annotated as membrane proteins. Moreover, most of these proteins are from *B. hyodysenteriae*; no proteins from *B. pilosicoli* have been described.

In the present work, we used a proteomic shotgun approach to characterize the proteins bound to the cell surface (proteomic surfaceome) and the subset of proteins present in the extracellular milieu (exoproteome; Desvaux et al., 2009; Armengaud et al., 2012) of *B. hyodysenteriae* and *B. pilosicoli*. To specifically describe the components of the exopeptidome, we also analyzed cell media extracts that were not treated with protease. Because our analytical conditions do not preserve cell viability and because the outer membrane of this species is labile, it can be expected that many of these exogenous peptides and proteins potentially derive from cell leakage or from the periplasmic space rather than from active cellular mechanisms. Still, their characterization can be important to determine possible factors involved in the pathogenicity and recognition of these species. In these experiments, we sought to gather the greatest possible coverage of the detectable proteome by performing multiple biological replicates and including several strains of *Brachyspira*.

## Materials and methods

### *Brachyspira* strains

Three isolates of *B. hyodysenteriae* (INFE1, V1, LL1) and two isolates of *B. pilosicoli* (OLA9 and Vi13) obtained from the Iberian pig breed in farms in central Spain (Extremadura and Castilla-León) were used in this study (Table 1). Strain identification was carried out by PCR using species-specific primers for nox (*B. hyodysenteriae*) and 16S rRNA (*B. pilosicoli*) (La et al., 2006).

**Table 1:** PCR characterization of the ***Brachyspira*** strains studied^*^.

**Strain**	**Pig breed**	**Region**	**Date**	**PCR**	***clpX***	***ftnA***	***ACP***	***bitC***	***tlyA***	***hlyA***	***nox_hyo_***	***smpA***	***smpB***	***vspF***
*INFE1*	Iberian Duroc	Badajoz	3/10/2009	*BRAHW*		X	X	X	X	X	X		X	X
*V1*	Iberian	Badajoz	10/16/2008	*BRAHW*		X	X	X	X	X	X	X		X
*LL1*	Iberian Duroc	Llerena	11/6/2009	*BRAHW*		X	X	X	X	X	X	X		X
*Vi13*	Iberian	Salamanca	11/17/2011	*BRAPL*	X		X			X				
*OLA9*	Iberian	Badajoz	10/26/2011	*BRAPL*	X	X	X			X				

* *The isolates were classified on the basis of the nox and 16S rRNA genes (La et al., 2006). Data from San Juan (2015). Primers used for PCR amplification were those described in Barth et al. (2012)*.

### *Brachyspira* cultures

Culture media were based on those described by Calderaro (Calderaro et al., 2001, 2005). In addition, various antimicrobial agents to which *Brachyspira* bacteria are resistant were added to the culture medium to remove most of the fecal micropopulation (Feberwee et al., 2008). The medium was composed of blood agar base n°2 (40 g/L) supplemented with 5% defibrinated horse blood (50 mL; Oxoid, Thermo Scientific, Waltham, MA, USA), beef extract (3 g/L), Bacto-peptone (5 g/L; Difco, BD, Franklin Lakes, NJ, USA), and spectinomycin (0.2 g/L), spiramycin (0.025 g/l), rifampicin (0.012 g/l), vancomycin (0.0062 g/L), and colistin (0.00625 g/L; all from Sigma-Aldrich, St. Louis, MO, USA), and 810 mL distilled water. The plates were incubated for 4–7 days at 42°C in an anaerobic jar with H_2_ and CO_2_ produced by an AnaeroGen TM 3.5 L (Oxoid, Thermo Scientific, Waltham, MA, USA). The colonies were examined by contrast microscopy (40x).

To obtain sufficient mass for analysis, solid subcultures in blood agar were used as the starting material. Samples were seeded in BHI medium (Laboratorios Conda, Pronadisa Torrejón de Ardoz, Spain) enriched with horse serum (15%) and incubated with stirring in anaerobiosis jars at 42°C for 4–7 days.

The cells were recovered by several centrifugations in 50 mL tubes at 12,900 × g for 10 min. The cell pellets were washed three times with TE buffer (10 mM Tris, pH 8.0, 1 mM EDTA, both from Sigma-Aldrich, St. Louis, MO, USA).

### Sample preparation

Bacterial pellets were resuspended in PBS (Sigma-Aldrich, St. Louis, MO, USA), and aliquots of 1 mL were transferred to 1.5 mL Eppendorf tubes (Eppendorf AG, Hamburg, Germany). The samples were pelleted again by centrifugation at 13,800 × g for 1 min. The amount of material in each aliquot was determined by weight (23 mg per aliquot on average), and the samples were stored at −80°C until further analysis.

Two aliquots of each strain were used per experiment, one for the analysis of the surfaceome and one for the analysis of the exoproteome and exopeptidome fractions. Three independent experiments, each one comprising the preparation and proteomic analysis of these three cell fractions from the five isolates, as described below, were performed during this study (Figure S1).

For surfaceome analysis, the cell pellets were resuspended in 150 μL of 25 mM ammonium bicarbonate (Sigma-Aldrich, St. Louis, MO, USA), and 100 μL of 0.1 μg/μL trypsin (sequencing grade, Promega, Fitchburg, WI, USA) was added. The samples were incubated at room temperature for 1 h and subsequently harvested by centrifugation at 2000 × g for 20 min at 13°C. The supernatants were transferred to fresh tubes and incubated for 7 h at 37°C.

For exoproteome/exopeptidome extracts, the cell pellets were resuspended in 250 μL of 25 mM ammonium bicarbonate, incubated at room temperature for 1 h, and harvested at 2000 × g for 20 min at 13°C. The supernatants were transferred to fresh tubes. Two aliquots (100 and 90 μL) were obtained from each supernatant for the exoproteome and exopeptidome analyses, respectively. For exoproteome analysis, the samples were incubated for 7 h at 37°C after addition of 40 μL of 0.1 μg/μL trypsin (sequencing grade). Samples for exopeptidome analysis were incubated at 37°C for 7 h without addition of trypsin. Digestion was terminated by the addition of TFA (final concentration 1%), and the samples were stored at −40°C until LC-MS/MS analysis.

### Nano-LC-MS/MS

Aliquots of each extract corresponding to 2 mg of the original bacterial pellet were concentrated to approximately 5 μL and brought to 20 μL volume with 1% formic acid, 5% methanol. Depending on sample availability, each extract was injected at least in duplicate; in most cases, triplicate or quadruplicate samples were injected.

LC-MS/MS peptide analysis was performed using an Agilent 1200 nanoflow system (Agilent Technologies, Santa Clara, CA, USA) coupled to an LTQ-Orbitrap XL mass spectrometer (Thermo Fisher Scientific, Waltham, MA, USA) equipped with a nanoelectrospray ion source (Proxeon Biosystems, Odense, DK). The HPLC system consisted of an Agilent 1200 capillary pump, a binary pump, a thermostatted microinjector and a microswitch valve. A 12-cm long, 100-μm-I.D., 5 μm, C18 column (Nikkyo Technos Co., Bunkyo-ku, Tokyo, Japan) preceded by a C18 preconcentration cartridge (Agilent Technologies) was used.

Chromatography was performed at 0.4 μL/min (Solvent A, 0.1% formic acid in water; Solvent B, 0.1% formic acid in ACN) using a multisegment linear gradient of buffer B as follows: 3–10% in 9.5 min; to 40% in 170.5 min, to 90% in 1 min, and to 100% B in 5 min. Mass spectra (400–1800 m/z) were acquired in data-dependent acquisition (DDA) mode at a resolution of 60,000. The 10 most abundant ions in the linear ion trap were sequentially selected for sequencing by collision-induced dissociation, using collision energy of 35%. Ions already selected for fragmentation were dynamically excluded for 45 s. The spray voltage was 1.8 kV, and the heated capillary temperature was 200°C.

To control analytical performance along the study, each file was processed using Proteome Discoverer 1.4v (Thermo Fisher Scientific, Waltham, MA, USA) and the Uniprot Database *Brachyspiraceae* (August 2014, 28,436 entries). The resulting data were used to monitor the number of new peptides and proteins identified per run. The searches used the following parameters: Enzyme, trypsin allowing 1 missed cleavage (no enzyme for peptidome fractions); dynamic modifications, acetyl N-terminus, methionine oxidation, and asparagine and glutamine deamidation. Precursor mass tolerance was set to 20 ppm and fragment mass tolerance to 0.8 Da. For database search and False Discovery Rate (FDR) calculation, Proteome Discoverer uses a decoy database automatically generated from the target database. Filtering of the search results is carried out by the Proteome Discoverer tool Percolator. For this purpose FDR limits were set at 0.001 and 0.01 for the Percolator FDR strict and FDR relaxed parameters, respectively. Final results were filtered by protein filters (peptide rank 1 and 2 peptides per protein) and peptide filters (set to medium peptide confidence which corresponds to FDR < 0.005).

### Database search

PeptideShaker (version 1.6.0; Barsnes et al., 2011) was used for peptide and protein identification from the full MS data collection. Through its SearchGUI user interface (version 2.1.4), this application combines six different search engines: OMSSA (version 2.1.9; Geer et al., 2004), Amanda (version 1.0.0.5242; Dorfer et al., 2014), X-tandem! (version 2013.09.01.1; Bjornson et al., 2008), MS-GF+ (version Beta v10282; Kim and Pevzner, 2014), Comet version 2015.02 rev.1 (Eng et al., 2013), and MyriMatch version 2.2.140 (Tabb et al., 2007). Searches were carried out against concatenated target/decoy versions of the Uniprot Databases for *Brachyspiraceae* and for *B. hyodysenteriae* and *B. pilosicoli* (all from November 2015, 40,573, 14,301, and 7670 entries, respectively). The decoy sequences were created by reversing the target sequences with SearchGUI.

Search parameters included acetyl N-terminus, methionine oxidation, and pyrrolidone from glutamic, glutamine, and carbamidomethylated cysteine as dynamic modifications. Precursor mass tolerance was set to 20 ppm and fragment mass tolerance to 0.6 Da. Surfaceome and exoproteome samples were searched setting trypsin as the enzyme and allowing two missed cleavages. Peptide analyses were carried out with no enzyme set.

After database search, PeptideShaker uses protein inference algorithms for protein characterization. Peptide Spectrum Matches (PSM), peptides and proteins were validated at 1% FDR estimated using the decoy hit distribution. Post-translational modification localizations were scored using the D-score (Vaudel et al., 2013) as implemented in the compomics-utilities package (Barsnes et al., 2011).

The mass spectrometry data, along with the PeptideShaker identification results, have been deposited to the ProteomeXchange Consortium (Vizcaíno et al., 2014) via the PRIDE partner repository (Martens et al., 2005) with the dataset identifier PXD003900.

### Data analysis

MS sequencing data were fed to PeptideShaker grouped by strain and cell fraction, originating a total of 15 PeptideShaker output files. PeptideShaker reports for peptide and protein identifications were exported to MS Excel format; from there, the data were read, combined, and further processed using Python scripts. Total PSM in the samples were normalized taking into account the corresponding number of replicates for each sample. For searches in the full *Brachyspiraceae* database, when different accessions of identical probability were assigned for an entry, the first accession corresponding to the species studied was selected as the group head. To obtain the list of surfaceome-specific proteins, the peptides, or proteins identified in the cell supernatants (exoproteome) were subtracted from those identified in the surfaceome sample. For this, a peptide or protein was considered to be present in a compartment when it represented more than 5% of the total counts for the three compartments and the two species. Otherwise, its presence was suspected to result from analytical or non-specific compartment cross-contamination. To compare the protein collections between species, proteins were indexed by their Uniprot names. For this purpose, Uniprot names were standardized by applying a group of simple rules (elimination of commas, hyphenations, etc.) to facilitate the comparison of identical protein names with small differences in their database annotations.

The properties of the protein collections were described using Gene Ontology Annotation (GOA), obtained through STRAP v1.5 annotation software, and freely available prediction software (LipoP v1.0, SignalP v4.0, PSORTb v3.0.2). LipoP v1.0 detects putative lipoproteins in Gram-negative bacteria and predicts the cleavage site of the signal peptide. This software has been trained on SPaseI-cleaved proteins, lipoproteins (SPase II-cleaved), and cytoplasmic and transmembrane proteins and is able to assign proteins to one of these classes on the basis of the protein's N-terminal sequence (Juncker and Willenbrock, 2003). SignalP v4.0, which detects potential signal peptides using neural networks, was designed to discriminate between signal peptides and transmembrane regions (Petersen et al., 2011). PSORTb predicts protein subcellular location in Gram-negative bacteria, classifying proteins according to five major locations (cytoplasmic, inner membrane, periplasmic space, outer membrane, and extracellular; Yu et al., 2010).

Hierarchical clustering of the peptides detected in the different strains was performed using GENE-E software version 3.0.204 (http://www.broadinstitute.org/cancer/software/GENE-E/). The column and row distance metric was one minus Pearson's correlation, and an average linkage method was used.

### Ethical statement

No animals were housed, infected, or pharmacologically treated for the study. Data presented was obtained from laboratory-grown bacterial strains. Original bacterial strains conserved at the cell bank were isolated from pig feces provided by field veterinarians carrying out their routine activity.

## Results and discussion

### Shotgun proteomics characterization

We applied a shotgun proteome approach to the study of the surfaceome, exoproteome, and exopeptidome of two *B. pilosicoli* and three *B. hyodysenteriae* strains, aiming to obtain the highest possible coverage of the host-exposed proteome of these *Brachyspira* species.

**Figure 1:**
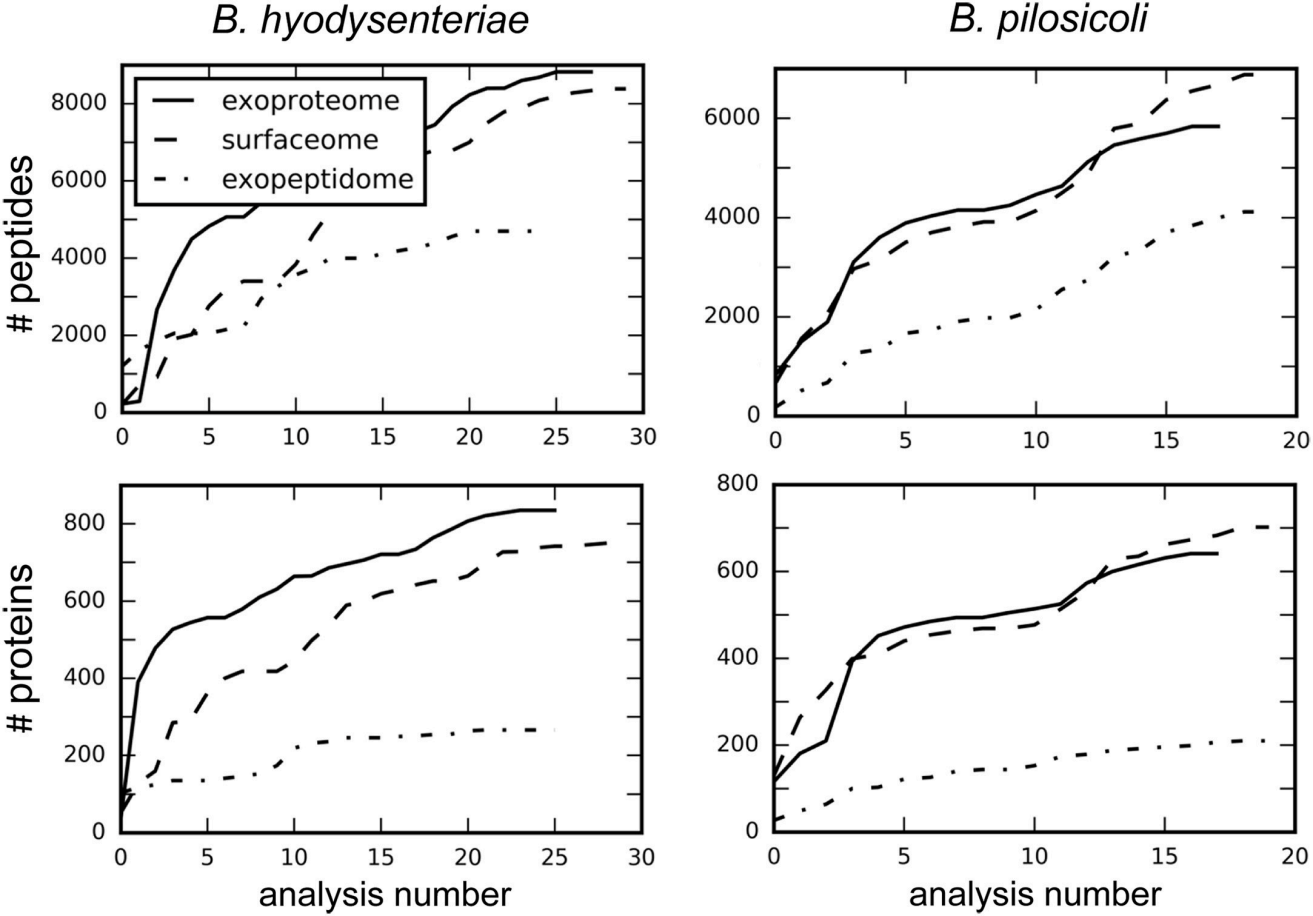
**Increase in the total number of identified peptides and proteins with the number of combined analyses**. Values were obtained by searching each independent file with Proteome Discoverer 1.4.

Despite recent advances in the field of discovery proteomics, comprehensive characterization of proteomes is still a major challenge, and requires extensive resources in terms of time, sample amount, and instrumentation (Gstaiger and Aebersold, 2009). Thus, no study of proteome mapping has reached 100% coverage of the proteins predicted from a genome (Ahrens et al., 2010). In addition to biological factors (i.e., lack of expression of some proteins under some conditions), limitations of the current MS instrumentation for the shotgun approach, in terms of scan speed and sensitivity, determine the degree of coverage obtainable from one analysis. As a consequence and due to the mechanics of data-dependent MS analysis, repeated analysis of the same sample provides different, partially overlapping protein collections. When sample amount is not limiting, combination of data from replicate experiments can increase the number of detected peptides, and proteins to a point determined by the detection limit of the technology. Following this strategy, in our study each sample was processed thrice and in each of these experiments the different fractions were analyzed by LC-MS/MS at least in triplicate. A total of 148 injections were carried out to reach the maximum coverage for each species and compartment (Figure 1).

**Figure 2:**
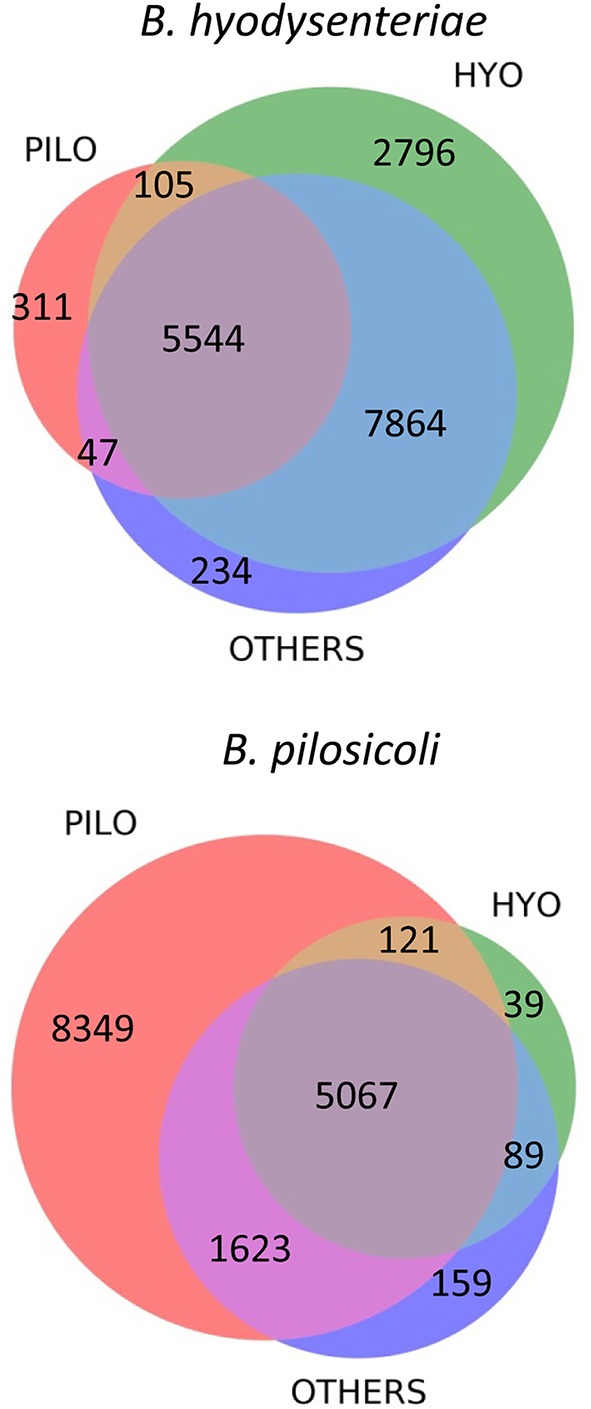
**Distribution of sequence matches in the different ***Brachyspira*** annotated proteomes for the collection of peptides identified in the ***B. hyodysenteriae*** and ***B. pilosicoli*** samples**. PILO, *B. pilosicoli*; HYO, *B. hyodysenteriae*; OTHERS, other *Brachyspira* species.

More than 29,000 different peptides were identified in the PeptideShaker search of the MS/MS data against the complete *Brachyspira* database (Tables S1–S3). This database contains mainly annotations from the proteomes of *B. hyodysenteriae, B. pilosicoli*, “*B. hampsonii*,”* B. intermedia, B. suanatina*, and *B. murdochii* (88% of all annotations) and a few sequences from other species such as *B. innocens, B. alvinipulli*, “*B. canis,”* and “*B. corvi”*. Overall, 16,970 and 15,493 peptide sequences were identified in *B. hyodysenteriae* and *B. pilosicoli*, respectively. Most of these peptides corresponded to the sequences expected from the annotated proteome of the respective species. Approximately 1.9% (*B. hyodysenteriae*) and 3.5% (*B. pilosicoli*) of the peptide matches in each of these collections, however, corresponded to sequences from proteomes other than those of the species analyzed (Figure 2). These figures are higher than that of the FDR for these collections (<1% at the peptide level). The origin of these trans-species matches is likely diverse. Matches of spectra from *B. pilosicoli* samples with *B. hyodysenteriae* sequences and vice versa can derive from residual contamination of the analytical system. Other trans-species-only matches may result from errors in assignation by the search engines, errors in database annotations, or even from a lack of the corresponding protein sequence in the database of the analyzed species. In fact, a previous study of the *B. pilosicoli* data using the smaller database version of 2014 produced approximately 8% trans-species-only matches, of which nearly 30% fully matched *B. pilosicoli* proteins in the current, more complete database (not shown). Trans-species-only matches could also reflect genetic or transcriptional differences between our strains and those used as the source of the Uniprot annotations, which include 14 different strains of *B. hyodysenteriae*, with strain ATCC 49526 as a reference proteome, and the *B. pilosicoli strains* ATCC BAA-1826, B2904, P43/6/78, and WesB. To test these hypotheses, we performed a BLAST analysis on a random sample of 100 peptides detected in *B. pilosicoli* samples that matched sequences from other species. More than half of these sequences (54%) showed more than 80% amino acid identity with the sequences of the studied species (differences in 1–3 amino acids, depending on the peptide size). In a significant proportion of cases in this group, the observed differences between the alternative sequences could be explained by a single nucleotide change. The four most common substitutions, D/N, I/V, K/R, and Q/K, represented more than 40% of the total observed differences. Assignment to other species due to Q/K differences most probably results from search engine limitations associated with assignment to the isobaric counterpart in the case of L and I or Q and K. D/N substitutions imply a difference of one mass unit between the alternative sequences that can also produce search engine mis-assignments. This can also be the case in combinations of two or more amino acid changes, which may produce isobaric sequences, or sequences with small mass differences. Leaving aside these possible assignment errors, the other cases observed support the idea that some of these trans-species-only matches are a reflection of both strain variability, and the still incomplete annotation/curation of the available *Brachyspira* databases.

The sequences of many of the peptides identified in *B. hyodysenteriae* were conserved in the proteome of *B. pilosicoli* and other species (80% of common peptides). In the case of *B. pilosicoli*, only 44% of the identified peptides were common to other species, whereas 54% of the sequences were *B. pilosicoli*-specific. This characteristic of the *B. pilosicoli* collection of sequences is consistent with differences observed in the comparison of several species of *Brachyspira* by MALDI-TOF protein profiling (Calderaro et al., 2013). Hierarchical clustering of the MALDI profiles produced a dendrogram in which the different species were located in two major branches. *B. hyodysenteriae, B. murdochii*, and *B. intermedia* clustered in different sub-branches of one of the major branches, whereas *B. pilosicoli* and *B. alborgii* were located in the other. Similar differences are observed in phylogenetic trees constructed from the 16S rRNA genes of several species of *Brachyspira* (Wanchanthuek et al., 2010).

A similar number of peptides was identified in the surfaceome and exoproteome fractions. In comparison, the number of exopeptidome peptides in the exopeptidome was lower (Figure 1, Table S2). When considering the number of assigned proteins the difference between the exopeptidome and the other two fractions increases. This could be due in part to the different composition of the digested and non-digested fractions (peptides vs. proteins) and the bias induced by our filtering protocols. Thus, for the Proteome Discoverer searches used to monitor individual analyses, the results of which are depicted in Figure 1, the identification of at least two different peptides per protein was required for a protein to be considered identified. This is a reasonable filter for the surfaceome and exoproteome collections but produces an underestimate of the actual confident identifications in the exopeptidome analysis, in which only small individual peptides are susceptible to being identified. PeptideShaker, which was used for the analysis of the full data, does not include the two-peptide requirement for protein inference; therefore, differences between the yield of peptides and proteins in the different compartments are smaller.

On average, ca. 300 proteins were identified per run in the surfaceome and exoproteome, and ca. 80 proteins were identified in the exopeptidome fractions. Due to the low degree of overlap of the identifications obtained between analyses, the total number of identifications of peptides and proteins increased rapidly for the first replicates (Tabb et al., 2010). This speed steadily decreased with the number of analyses performed, although a plateau was not clearly reached even after more than 40 analyses (Figure 1).

Considering all fractions, the PeptideShaker protein inference analysis using 1% FDR at all levels (PSM, peptide, and protein) pointed to 1625 and 1338 different protein accessions in *B. hyodysenteriae* and *B. pilosicoli* samples, respectively (Tables S4–S6). To facilitate large-scale analysis of the functional information derived from these data, for some studies we indexed the collections by gene, and protein name instead of by accession. Grouping by protein name has the disadvantage of including in the same group proteins with completely different sequences but with a common protein name. In return, grouping by gene or name tends to eliminate the redundancy derived from the existence of protein isoforms and homologous or identical protein sequences identified in different strains, sequencing projects that are present in the current *Brachyspira* databases. Overall, 2963 protein accessions were identified among the three fractions and the two species analyzed (surfaceome and exoproteome/exopeptidome analysis, non-redundant data). When indexed by gene and name, this figure was reduced to 1793 entries (1243 and 1060 for *B. hyodysenteriae* and *B. pilosicoli*, respectively). The genomes of these species have been described to contain 2153 (*B. hyodysenteriae*, WA1) and 1987 (*B. pilosicoli* 95/1000) protein-coding genes (Wanchanthuek et al., 2010). Current UniProt databases include 14 *B. hyodysenteriae* strains with an average of ca. 2640 protein sequences. Data for *B. pilosicoli* are fewer, restricted to four strains and still unreviewed. In this case, the largest database contains 2638 entries. Considering these figures, the number of accessions in our collection would roughly correspond to 62% (*B. hyodysenteriae*) and 50% (*B. pilosicoli*) of the proteins annotated for these species.

### Distribution by compartment

Peptidome and exoproteome samples were prepared from aliquots of the same cell supernatants that were analyzed directly (peptidome) or after tryptic digestion (exoproteome). Although the exoproteome samples contain all the peptides detectable in the peptidome samples, many of these peptides will be further hydrolyzed by trypsin, yielding sequences that are too small to be detected, or from which the original state of the peptide cannot be inferred. Thus, the exopeptidome analysis provides information on the low-molecular-weight components of the sample, including oligopeptides and protein hydrolysis products present in the supernatants. The surfaceome collection was prepared from the same cell aliquots using a cell shaving strategy. Cell shaving, which is based on limited proteolytic digestion of the whole bacterial cell, has been described as an efficient method for characterization of the cell surfaceome (Tjalsma et al., 2008; Doro et al., 2009; Solis et al., 2010; Gesslbauer et al., 2012). This method allows characterization of exposed outer membrane proteins and exposed sections of internal membrane proteins as well as extracellular proteins that could be bound to the membrane.

The 10 most abundant peptides detected in each compartment in terms of validated PSM (Table 2; complete data are provided in Table S2) point to a few proteins, including glyceraldehyde-3-phosphate dehydrogenase, flagellar filament proteins FlaB3, and FlaA1, elongation factor Tu, 60-kDa chaperonin, acyl carrier protein, rubrerythrin, thiol peroxidase, and NADH oxidase. These proteins are also among the 10 more abundant proteins in the collection of proteins inferred by PeptideShaker (Table 3).

**Table 2:** The 10 most abundant peptides identified in each compartment for ***B. pilosicoli*** and B. hyodysenteriae; Values correspond to the PeptideShaker validated PSM for each sequence^*^.

**Peptide**	***Hyodysenteriae***			***Pilosicoli***			**Protein**	**Accessions**	**Species**
	**pep**	**prt**	**surf**	**pep**	**prt**	**surf**			
EIDVVGVVDVSTDAK	5	0	3	0	1323	1140	G3PDH	D8IB86; J9UE83; K0JJQ2	BRAPL
DLGVEYVIESTGLFTDKEK	4	0	0	0	558	643			
DLGVEYVIESTGLFTDK	0	0	0	0	776	53			
ADITTEGEDVLVVNGNK	4	85	57	2	782	332		A0A0H0UCR6; C0R213; D8IB86; J9UE83; K0JJQ2; Q8VNZ1	C
AEGHIAAGAK	0	45	219	0	326	568			
EKAEGHIAAGAK	0	69	112	0	25	63			
EIEVVGVVDVSTDAK	0	262	94	0	0	0		A0A0H0UCR6; C0R213; Q8VNZ1	BRAHW
ALGVEYVIESTGLFTEK	0	132	96	0	0	0			
ALIQVEVNQLVAEVDR	0	308	94	0	841	80			
SLMIATENTIASESVIR	4	306	76	3	152	39			
INTAGDDASGLAVSEK	2	114	80	0	98	68	Flagellar protein FlaB3	A0A0H0USH8; C0R1D6; D8IDG1; J9UXQ6; K0JLS4; Q9F0F6	C
ELAIQSANGIYSDSDR	0	142	42	0	68	25			
IDEGIQMVVSQR	0	119	81	0	1	0		A0A0H0USH8; C0R1D6; Q9F0F6	BRAHW
NMITGAAQMDGAILVVSAEDGVMPQTK	2	85	78	0	534	364	ElongationFactor Tu	A0A0H0TPC4; C0QVZ4; D8ICZ6; J9UB96; K0JL99; P52854	C
TTLTSAITAVSSAMFPATVQK	0	79	99	0	7	14			
SLETSLSLVEGMQFDR	7	197	200	2	276	259	60 kDa chaperonin	A0A0H0TIS1; A0A0H0V6M8; C0QWM4; D8IB78; J9USS2; K0JLL7; Q3YLA1; Q3YLA3	C
AMLEDIAILTGGQVISEDLGMK	0	155	104	0	192	145		A0A0H0TIS1; A0A0H0V6M8; C0QWM4; D8IB78; J9USS2; K0JLL7	C
EVIITDIPEPEKPMPPMPGGGMGGMY	45	3	0	102	26	11			
ITDIPEPEKPMPPMPGGGMGGMY	36	7	3	92	30	14			
TAEVIITDIPEPEKPMPPMPGGGMGGMY	38	5	0	87	15	7			
VIITDIPEPEKPMPPMPGGGMGGMY	10	0	0	106	26	9			
ITDIPEPEKPMPPMPGGG	44	0	0	75	0	0			
FGPPTIINDGVTIAKE	2	0	0	77	22	4			
AKEIELEDPFENMGAQIVKEV	56	0	0	7	0	0			
DAIKLENPDEQVGVNIVKR	0	0	0	100	2	0		D8IB78; J9USS2; K0JLL7	BRAPL
ISNMKELLPILEK	44	11	17	0	0	0		A0A0H0TIS1; A0A0H0V6M8; C0QWM4; Q3YLA1	
TAELEDALLLIYDKK	43	9	6	0	0	0	60 kDa chaperonin		BRAHW
TVENPDEQVGVNIVK	40	9	5	0	0	0		A0A0H0TIS1; A0A0H0V6M8; C0QWM4	
LTVENPDEQVGVNIVKRAIEEPIRM	50	0	0	0	0	0			
ALIDEIKDVVANQLNISDK	0	113	63	2	548	188	Acyl carrier protein	A0A097BU34; C0QYL1; D8IBL1; J9USC6; K0JK36;O34163; Q6VAN3	C
FFEVRVESY	8	2	0	629	202	36	Rubrerythrin	D8ICG1; J9UBZ5; K0JKT4	BRAPL
KFFEVRVESY	0	1	0	79	68	49			
FEVRVESY	0	0	0	146	40	0			
AIIAEVFEEFASLSGR	0	0	0	0	427	267	Putative pyruvate oxidoreductase	D8ICR0; J9URY6; K0JMR2; O87445	BRAPL
AIILDVFEEFASLSGR	0	79	111	0	0	0		A0A0H0UY93; A0A0H0VE59; C0QV98	BRAHW
ITFQGGEVHLEGVSLVEGAK	1	0	0	0	483	194	Thiol peroxidase	D8IFX8; J9UV33; K0JFG9	BRAPL
AAGGGAQITAK	0	37	159	0	18	108	50S ribosomal L2	A0A0H0W8S2; C0QVZ9; D8ICZ1; J9UBA7; K0JL93	C
IKDAGIELHLGETVK	5	0	0	0	65	207	NADH oxidase	B1NIM3; D8IAM5; F1B291; G8DZP2; J9UGQ4; K0JKC2; Q7BTH4; Q9R903; Q9R904; Q9ZHJ2; S4UT40; T1W0C5; W0FBQ9	BRAPL
IREQAELR	0	72	146	0	0	0	Flagellar filament outer layer protein flaA1	A0A0H0TRE1; A0A0H0V489; A0A0H0WR23; C0R0T5; P32520	BRAHW
DADLIIEAAFENLEVK	0	207	0	0	0	0	3-hydroxybutyryl-CoA dehydratase	A0A0H0TSQ1; C0QWH3	BRAHW
DGVIQNVGLELIGEAK	0	51	97	0	0	0	Electron transfer flavoprotein beta subunit	A0A0H0UWP8; A0A0H0VH59; C0QV71	BRAHW
TLEYDIIISGR	0	20	97	0	0	5		A0A0H0U588; C0QV72; D8ID09; J9TR46; K0JMS4	C
IPGGEATPAPPLGPALGQKQ	40	2	0	39	3	0	50S ribosomal protein L11	A0A0H0XFL7; C0QWX2; D8IEH0; J9UXV3; K0JHR3	C
TINQKQLEEIAQEKMA	40	0	0	23	0	0			
RIPGGEATPAPPLGPALGQKQ	39	0	0	8	0	0			

* *The accessions column lists the accession number of all proteins containing the corresponding peptides. Exclusive indicates whether the corresponding protein was found in the samples from the two Brachyspira species (C) or was specific (>98% of total PSM) to one of them (BRAPL, BRAHW for B. pilosicoli, and B. hyodysenteriae, respectively). Full tryptic peptides were not considered for the peptidome list. Complete data is presented in Table S2*.

**Table 3:** **The 10 most abundant proteins identified in each compartment for ***B. hyodysenteriae*** and ***B. pilosicoli*****.

**Protein**	***Hyodysenteriae***			***Pilosicoli***		
	**Pep**	**Prt**	**Surf**	**Pep**	**Prt**	**Surf**
60 kDa chaperonin	5636	1817	1342	10564	2070	2168
Glyceraldehyde-3-phosphate dehydrogenase	146	1041	1164	2004	5077	4098
Flagellin	1708	2663	968	414	4531	1616
Rubrerythrin	650	457	325	1941	1133	578
Elongation factor Tu	155	881	762	120	1705	1210
NADH oxidase	25	892	958	42	1348	1464
Acyl CoA dehydrogenase	776	939	1527	183	294	967
50S ribosomal protein L7/L12	159	706	460	762	1329	1159
Alcohol dehydrogenase	40	476	174	148	2742	990
Pyruvate phosphate dikinase	38	600	1184	0	171	2476
Enolase	367	1155	722	67	1689	285
Pyruvate ferredoxin oxireductase	122	825	778	31	1772	1944
Flavoprotein	9	697	564	0	484	1391
Chaperone protein DnaK	11	979	379	70	802	422
Acyl carrier protein	25	278	168	182	1208	502
Pyruvate oxireductase	72	98	123	830	388	702
50S ribosomal protein L2	41	546	902	81	71	505
Methyl-accepting chemotaxis protein B	72	188	799	12	2	882
10 kDa chaperonin	21	285	144	1177	95	142
50S ribosomal protein L11	898	87	82	528	52	131
Flagellar filament outer layer protein flaA1	23	903	540	0	0	0
ATP-dependent 6 phosphofructokinase	691	151	102	41	226	60
Lipoprotein	1	97	860	15	140	145
2-isopropylmalate synthase	293	51	40	711	56	42
50S ribosomal protein L10	169	119	107	539	70	141
50S ribosomal protein L18	597	92	79	182	20	51
Rubrerythrin fusion protein	388	125	75	0	0	0

*Values correspond to the total validated PSM for all proteins in the described group. The complete data are presented in Table S5*.

Characterization of compartment-specific components from these collections is not straightforward, especially for the exoproteome and surfaceome components. The procedure for the preparation of the surfaceome sample is identical to that used for the exoproteome except that, in the former case, trypsin is already present during the first hour of incubation prior to cell elimination. Thus, the surfaceome sample contains both surface and exogenous proteins, and characterization of the surface-specific proteins requires subtraction of the exoproteome collection from that of the surfaceome. Determination of these differences is also complicated by the fact that the studied compartments are not hermetic. Proteins strictly considered membrane proteins can also be found in the culture medium due to cell lysis or contamination of the sample with cell debris, and elimination of these proteins would constitute a false negative for the surfaceome. Thus, as expected, most of the peptides and proteins identified in our experiments were detected in several compartments, although in some cases this was supported by a high number of validated peptide sequence matches. Because peptide sequence matches can be considered a rough estimate of protein abundance, we used this parameter to distinguish tentative compartment-specific proteins from proteins derived from compartment cross-contamination. For this, we filtered out from each compartment all peptides and proteins that were supported by fewer than 5% of the total peptide sequence matches pointing to them in the three fractions and considered only peptides and proteins with a minimum number of validated peptide spectra (5 and 15 for peptides and proteins, respectively).

**Figure 3:**
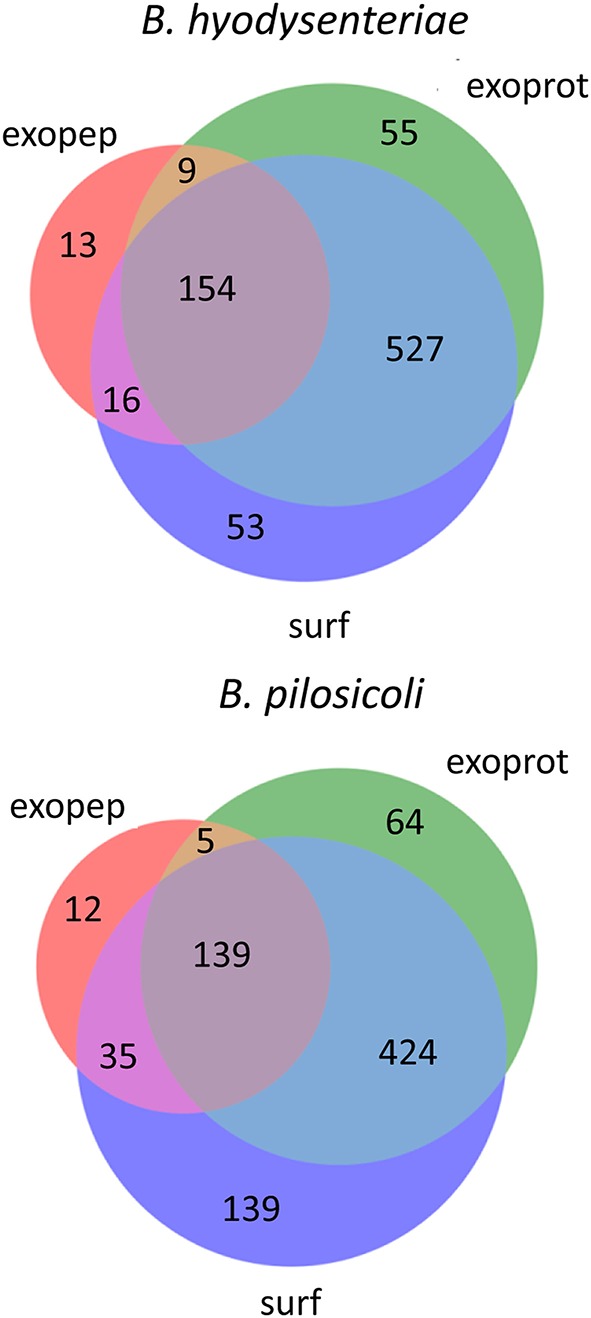
**Distribution of identified proteins (unique accessions) among compartments**. Only proteins with more than 15 validated PSM are shown in the figure. Proteins considered exclusive must have more than 80% of total validated spectra in one compartment, and any other compartment, considering each species separately, must contain <5% of the total validated spectra.

Using the above criteria, 192 accessions from *Brachyspira* proteins (53 and 139 for *B. hyodysenteriae* and *B. pilosicoli*, respectively) were classified as surfaceome-specific, whereas 119 were classified as specific to the exoproteome sample (55 and 64 for *B. hyodysenteriae* and *B. pilosicoli*, respectively; Figure 3).

Whereas the total number of *B. pilosicoli* proteins is slightly lower than that of *B. hyodysenteriae*, the number of proteins identified as exclusive to the surfaceome in *B. pilosicoli* is nearly triple that found in *B. hyodysenteriae*. This difference between species, which is observed independently of whether the collections are indexed by name or by accession and at any PSM cutoff, could reflect higher resistance of *B. hyodysenteriae* to trypsin hydrolysis. In fact, the different composition of the outer membrane LOS (Lipooligosaccharides) of these species makes the *B. pilosicoli* membrane more easily disruptable than the membrane of *B. hyodysenteriae* (Trott et al., 2001).

### Surfaceome

**Figure 4:**
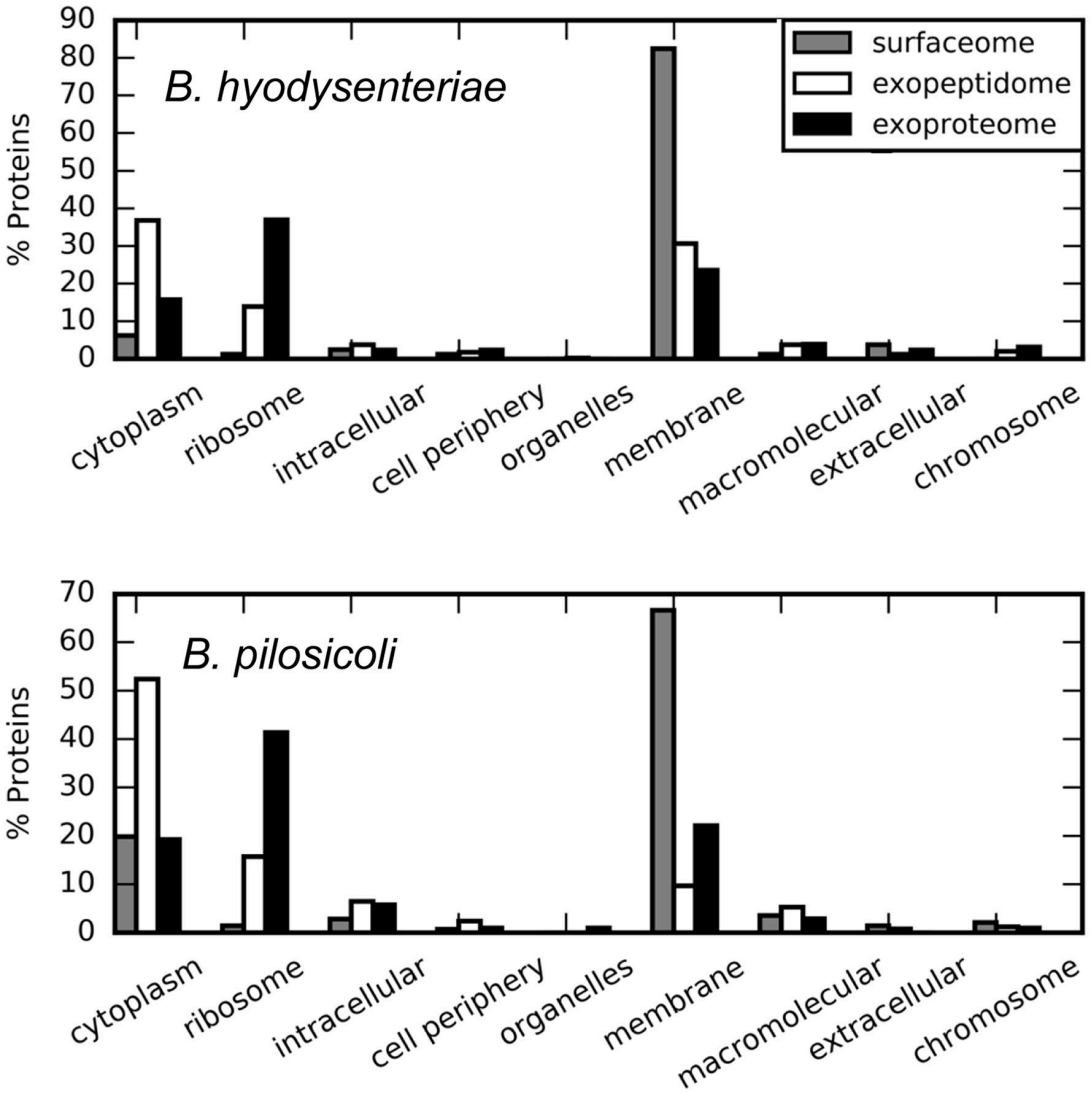
**Cellular location of the compartment-specific proteins obtained using STRAP**. GO annotation, Cell component.

The most abundant of the surfaceome-specific proteins, in terms of validated PSM, are indicated in Table 4 (the full collection, indexed either by accession or name, is provided in Table S5). Among the proteins with GO annotation, more than 60% of the surfaceome-specific proteins are likely located on the membrane, and an additional small fraction is defined as extracellular or located in the cell periphery (Figure 4). This enrichment in membrane proteins is also observed using the PSORTb prediction tool for bacterial protein location (Figure S2). In this case, nearly 50% of the proteins with predicted location are classified as from the periplasmic space, inner membrane or outer membrane, whereas another 10% of all proteins could have multiple locations. In contrast, near 90% of proteins in the exoproteome fraction with predicted location by PSORTb are cytoplasmic. According to LipoP predictions for a total of 184 surfaceome-exclusive proteins from *Brachyspira* (>2 PSM), 91% were classified as lipoproteins (SPaseII-cleaved proteins), SPaseI-cleaved, or transmembrane proteins (Figure S3). Considering only high-confidence assignations (margin > 4, 114 proteins assigned), 57 corresponded to lipoproteins, 44 to transmembrane, and 13 to proteins with SpI signal (Table S7).

**Table 4:** The 20 most abundant proteins specifically located in the surfaceome^*^.

**Protein**	**PSM**	**Accessions**	**Genes**	**Species**
Ribonuclease Y	582	A0A0H0V3X2, D8IB75, A0A0H0U8G9, C0R0C9	rny	C
DNA-directed RNA polymerase omega subunit family protein-like protein	322	D8IA88, K0JKN7	BP951000_2245, WESB_1288	BRAPL
Apolipoprotein A1/A4/E domain-containing protein	306	J9ULC7	B2904_orf1166	BRAPL
N-acetylglucosamine-1-phosphate uridyltransferase	203	C0QY93, D8IB64	glmU	C
Lon protease	169	D8ICT7, C0R248, J9URW0	lon	C
Preprotein translocase YajC subunit	135	D8IEE4	yajC	BRAPL
ATP synthase subunit b	128	C0QW62, D8IBP4	atpF	C
UPF0365 protein BP951000_0575	120	D8IBQ3	BP951000_0575	BRAPL
Unchar D8ICF3	114	D8ICF3	BP951000_0828	BRAPL
Phosphomannomutase phosphoglucomutase	112	D8I9Z9	manB	BRAPL
Transposase	106	K0JKA8	WESB_1932	BRAPL
Inositol-1-monophosphatase	98	C0QX44, D8IBH8	suhB	C
Unchar D8IEP8	90	D8IEP8	BP951000_1640	BRAPL
PTS system glucose subfamily IIA subunit	90	D8IBR5	ptsG	BRAPL
PTS system fructose-specific IIABC component	88	D8ID87	fruA	BRAPL
Transcriptional regulator XRE family	86	J9TSW8	B2904_orf609	BRAPL
PTS system fructose specific transporter subunit IIABC	83	K0JLH4	WESB_2470	BRAPL
Methyl-accepting protein	80	D8IBS9, C0R060	BP951000_0603, tar5	C
Unchar J9UB61	79	J9UB61	B2904_orf310	BRAPL
Transcriptional regulator CarD family	71	D8ID15	carD	BRAPL

* *The members column lists protein accessions identified by PeptideShaker contributing to the total validated PSM. Note that not all accessions individually fulfil the filtering criteria. Species indicates whether the corresponding protein was found in the samples from the two Brachyspira species (C) or was specific (>98% of total PSM) to one of them (BRAPL, BRAHW for B. pilosicoli, and B. hyodysenteriae, respectively). When PSM are lower than 50, species specificity is not determined (X) except when all counts belong to a single species and there are at least 25 PSM. Complete data is presented in Table S5*.

Approximately 5 and 20% of the surfaceome-specific proteins are classified as cytoplasmic according to GO annotation for *B. hyodysenteriae* and *B. pilosicoli*, respectively. As discussed above, the presence of cytoplasmic proteins in the extracellular compartments can be explained as the product of cell lysis (Christie-Oleza and Armengaud, 2010; Christie-Oleza et al., 2012). Gram-negative cells have been reported to be less resistant to the shaving process, with a higher level of cell death (Grandi, 2010), a characteristic that would be in agreement with an increased amount of cytoplasmic proteins. To reduce the contribution of possible leakage proteins, the surfaceome specific collection was obtained by subtracting the proteins detected in the exoproteome from the original surfaceome data. Thus, the presence of cytoplasmic proteins in our surfaceome-specific collection could also reflect the genuine presence of some of these proteins on the surface of the cells. In any case, only a small fraction of the proteins classified as cytoplasmic by GOA or the PSORTb prediction tool in the surfaceome-exclusive collection are predicted to bear a signal peptide-cleavage site by LipoP. This suggests that their presence on the cell surface, if genuine, would result from non-canonical transport mechanisms.

**Figure 5:**
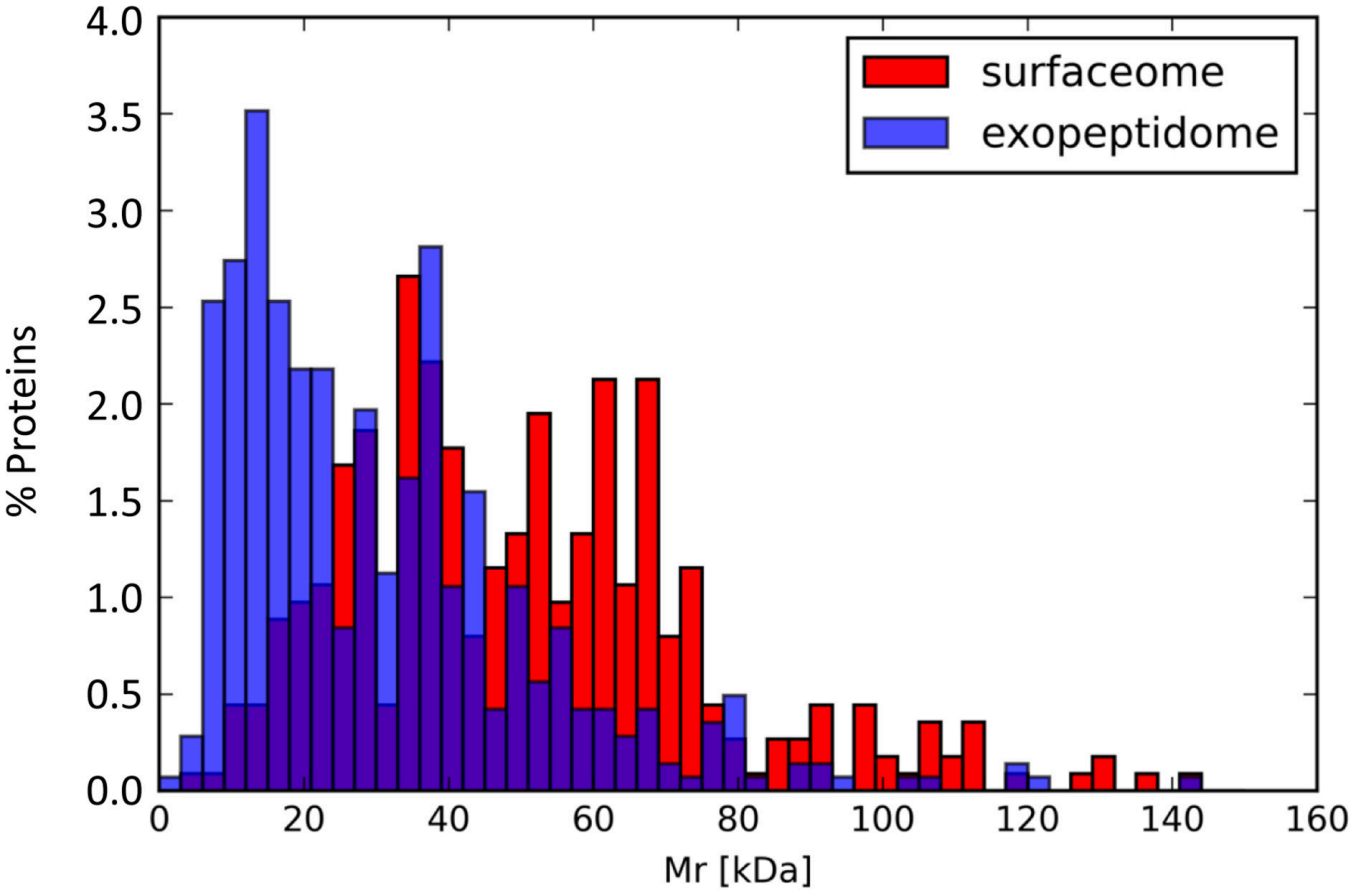
**Molecular mass distribution for the proteins annotated in the exopeptidome compared with those specific to the surfaceome**.

**Figure 6:**
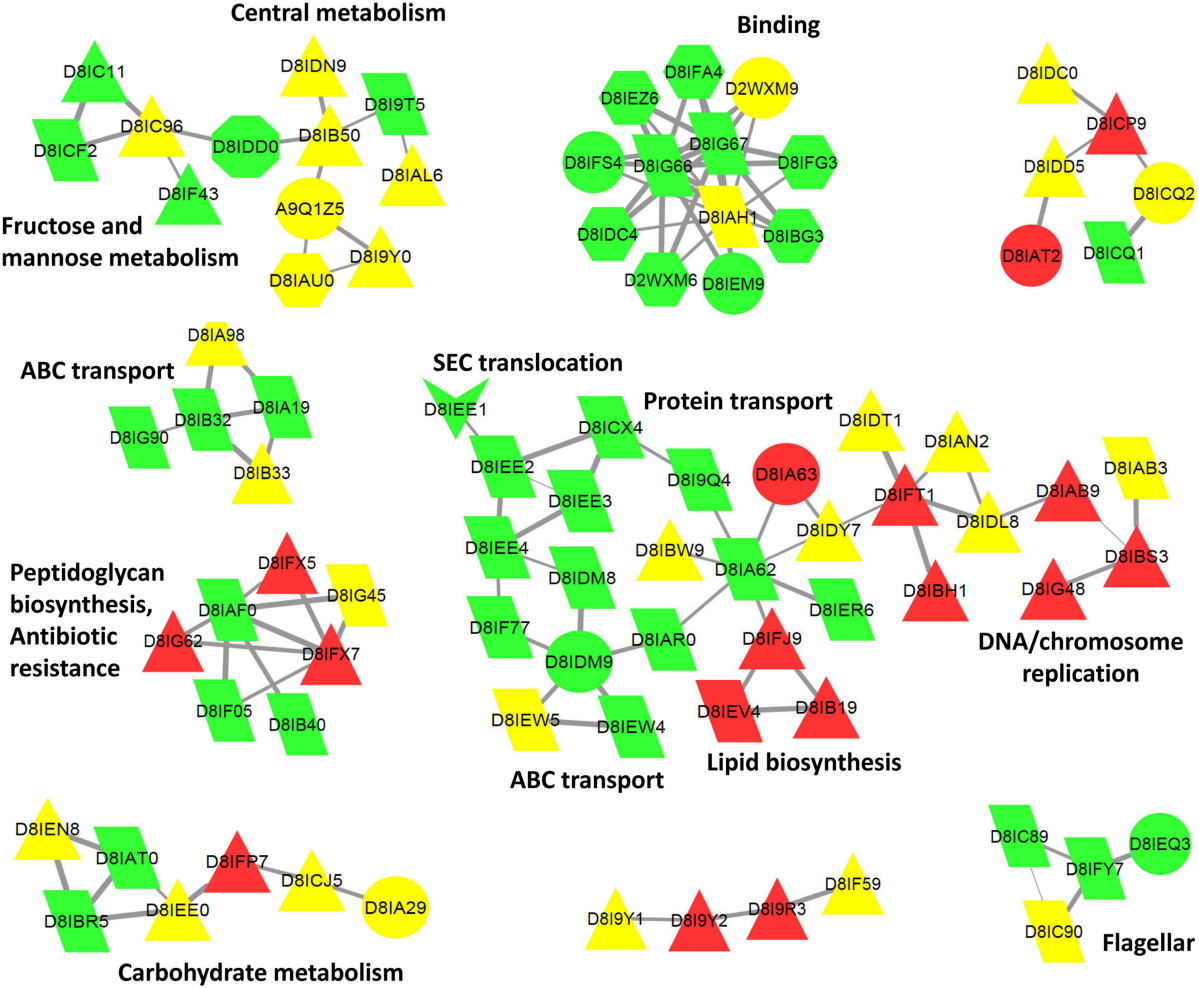
**Cytoscape biological network 47 of ***B. pilosicoli*** surface-exclusive proteins**. GOA Cellular Component Annotation (node color): green, membrane, and cell periphery; red, cytoplasm, and intracellular; yellow, unknown. PSORTb prediction (node shape): triangle, cytoplasmic; trapezoid, cytoplasmic membrane; octagon, outer membrane; hexagon, periplasmic; arrow, extracellular; circle, unknown. STRING Database: *B. pilosicoli*, Interaction Score ≥0.8.

Surfaceome-specific proteins display a wide range of molecular weights (Figure 5) and predicted isoelectric points. According to GO annotation, these proteins are involved in many different cell functions (Figure 6 and Figure S4). Many of them (ca. 48%) are classified as binding proteins, and a high proportion are transferases, hydrolases, or have some catalytic activity, with an overrepresentation of signal transducers and transporters relative to the peptidome or exoproteome.

### Exoproteome and exopeptidome

A total of 2053 proteins (non-redundant) were identified in the external proteome of *B. hyodysenteriae* and *B. pilosicoli* strains studied in this work. In contrast with the surfaceome collection, a high proportion of these proteins are annotated as cytoplasmic or ribosomal by GOA (Figure 4). PSORTb predictions assign a cytoplasmic location to approximately 81% of all proteins with known location (Figure S2). Specially, in the case of the *B. pilosicoli* exoproteome, cytoplasmic proteins account for more than 86% of these proteins.

According to LipoP predictions, 263 of a total of 301 exoproteome proteins from the two analyzed *Brachyspira* species (>2 PSM) were classified as lipoproteins (SPaseII-cleaved proteins), SPaseI-cleaved, or transmembrane proteins (see Table S8 for LipoP, PSORTb, and SignalP predictions for this collection). In contrast to the surfaceome-strict collection, in which transmembrane helix assignations were more frequent (42%, no margin cutoff) and only 17% showed an SpI signal, SpI proteins represented up to 34% of the proteins in the exoproteome fractions and only 17% of TMH assignations (Table S8).

Taking into account the protocols used in the preparation of the different samples, the protein collection from the exoproteome fraction would be expected to be included in that of the surfaceome compartment. Nevertheless, 119 protein accessions, representing nearly 9% of the total exoproteome assignations, were identified in *Brachyspira* samples as exoproteome-exclusive with high confidence (>15 PSM, Table 5). Due to the random character of the data-dependent MS scanning method, it is possible for some proteins with low numbers of validated peptide matches to be detected in one compartment and missed in another compartment in which they are also present. However, a number of exoproteome-exclusive proteins with high numbers of validated spectra (>100 PSM) were detected, making their presence in this category a result of limited spectral count statistics unlikely (Table 5). A tentative explanation for these identifications could be related to protein or peptide loss during the preparation of the surfaceome extract. In this process, trypsin is added to a cell suspension from which the cells are removed after 1 h. Some of the digested peptides produced from proteins in the medium could be lost in this step due to adsorption to the cellular pellet. In contrast, during the preparation of the exoproteome fraction, the cells are removed prior to digestion, and these losses may not occur, potentially resulting in the false positive identification of some peptides in the exoproteome fraction as exclusive.

**Table 5:** **Top 10 exoproteome-exclusive and exopeptidome-exclusive proteins (Table 4 for details)**.

**Protein**	**PSM**	**Accessions**	**Gene**	**Species**
**EXOPROTEOME**				
Superoxide dismutase	937	A0A0H0V406, C0QW83, D8ICG2	SR30_05435, sodA	C
Endoribonuclease	363	D8IFH7, C0R0L6	BP951000_1922, BHWA1_01174	C
Unchar D8IB10	248	D8IB10	BP951000_0328	BRAPL
Unchar D8I9Q8	195	D8I9Q8	BP951000_0016	BRAPL
CoxL protein	146	J9U0I3, K0JKH7	coxL	BRAPL
Variable surface protein VspD	144	D8ICU0, J9UQJ0	vspD, B2904_orf389	BRAPL
Unchar J9UGA5	122	J9UGA5	B2904_orf1414	BRAPL
Unchar J9U2W9	116	J9U2W9	B2904_orf2565	BRAPL
Flagellar filament protein FlaA	105	A0A0H0UUF0	SR30_02310	BRAHW
Nitroreductase	96	D8IFL9	BP951000_1964	BRAPL
Phosphate ABC transporter phosphate binding protein	94	D8IAV1, C0QY26	ptsS	C
Yqi (Fragment)	86	A9Q1Z1	yqi	BRAPL
Peptidase T	83	D8IFQ9, J9TSR4, C0QV99, K0JHK6	WESB_0904, pepT	C
Cytoplasmic protein	77	J9UX05	B2904_orf2396	BRAPL
**EXOPEPTIDOME**				
Rod shape-determining protein MreB	162	A0A0H0TUE9	SR30_05720	BRAHW
Electron transfer flavoprotein subunit alpha	98	A0A0H0VH59	SZ51_09315	BRAHW
Ferric uptake regulator	59	D8IDK7	fur	BRAPL
Ferredoxin 4Fe 4S	40	D8IA49	BP951000_2206	BRAPL
Phage terminase large subunit	26	D8IE54	xtmB	BRAPL
Pyruvate ferredoxin/flavodoxin oxidoreductase	23	D8IED5	porA	X
Unchar D8IDT5	20	D8IDT5	BP951000_1320	X
Unchar C0QVB6	19	C0QVB6	BHWA1_01956	X
Fer2/BFD BFD like 2Fe 2S binding domain-containing protein	19	J9UVB4	B2904_orf1553	X
Enzyme of poly gamma glutamate biosynthesis (Capsule formation)-like protein	18	C0QYZ6	BHWA1_00588	X

Although exoproteome/exopeptidome proteins display a high degree of overlap (Figure 3), some proteins were only identified in the exopeptidome samples (Table 5). Because the exopeptidome samples consist of cell supernatants that were not treated with trypsin, only small polypeptides of less than ca. 3–4 KDa are expected to be detected. In addition to protein fragments resulting from protein degradation, this compartment potentially includes bioactive peptides that either leak from or are actively secreted by the cells and are important for the interaction of the bacterium with its environment. It is interesting to note that the size distribution of proteins pointed to by the peptidome peptides shows a much higher proportion of molecules <10–20 KDa in size than the proteins specific to the surfaceome fraction, which have an average size of ca. 50 KDa (Figure 5), or those specific to the exoproteome (not shown).

**Figure 7:**
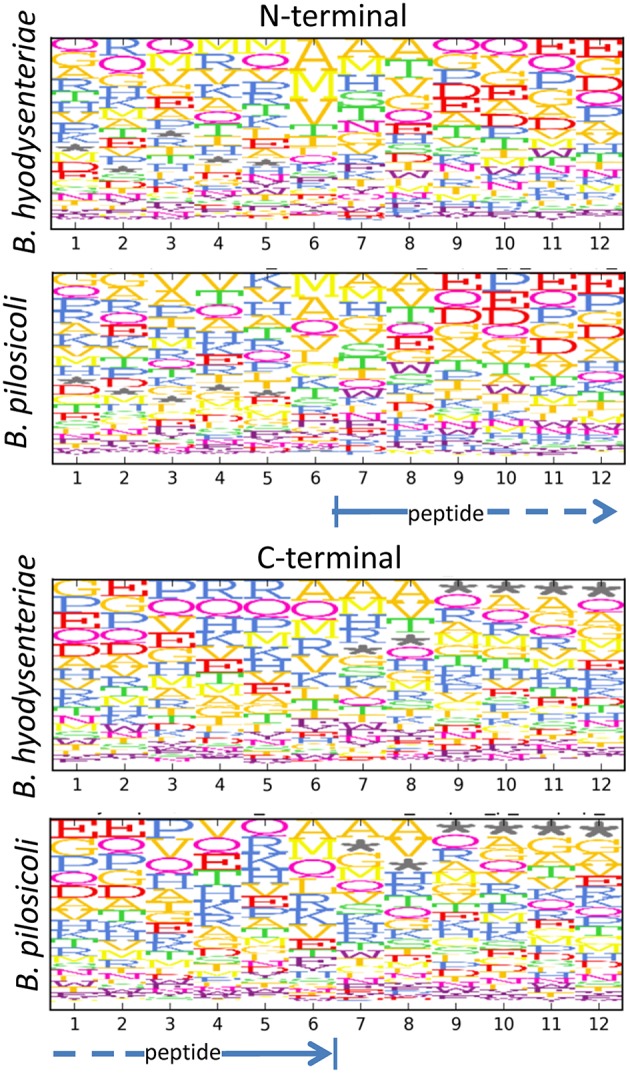
**Motifs in the N- and C-terminal regions of peptides in the exopeptidome fraction**. Size and top to bottom position of the amino acid letters are related with the frequency at which the amino acid is observed on that position. Frequencies are corrected for the amino acid frequency in the full *Brachyspira* database. An asterisk denotes the absence of an amino acid (off-sequence positions in protein terminal peptides).

When considering the peptides identified with a higher number of PSM in our collection (Table 2), it can be observed that many of these highly abundant peptides constitute groups of nested sequences of varying lengths. This is the case for three peptides with the common core FEVRVESY from rubrerythrin and many of the sequences from the 60 KDa chaperonin. In trypsin-treated samples, the occurrence of nested sets is primarily due to incomplete digestion of the samples, which produces sequences with missed cleavages. In the case of the peptidome samples, which were not treated with trypsin, these sets may reflect the activity of cell proteases present in the media. The analysis of the N- and C-terminal amino acid sequences of these peptides seems to indicate a slight enrichment of some amino acids, such as alanine and glutamine, in the peptide terminal sides but, apart from this, no clearly recognizable specific peptidase motif could be detected (Figure 7). The frequency of methionine in the N-terminal region (positions 6–7 in Figure 7) and that of off-sequence positions (indicated by an asterisk in Figure 7) in the C-terminal region suggested that many of the observed peptidome sequences correspond to C- and N-terminal peptides that have been further processed by the action of exopeptidases.

### Comparison between species and strains

Several differences between *B. pilosicoli* and *B. hyodysenteriae* have already been mentioned, including the significantly greater number of species-specific peptide matches in *B. pilosicoli* and the higher proportion of surfaceome-exclusive proteins in this species.

**Figure 8:**
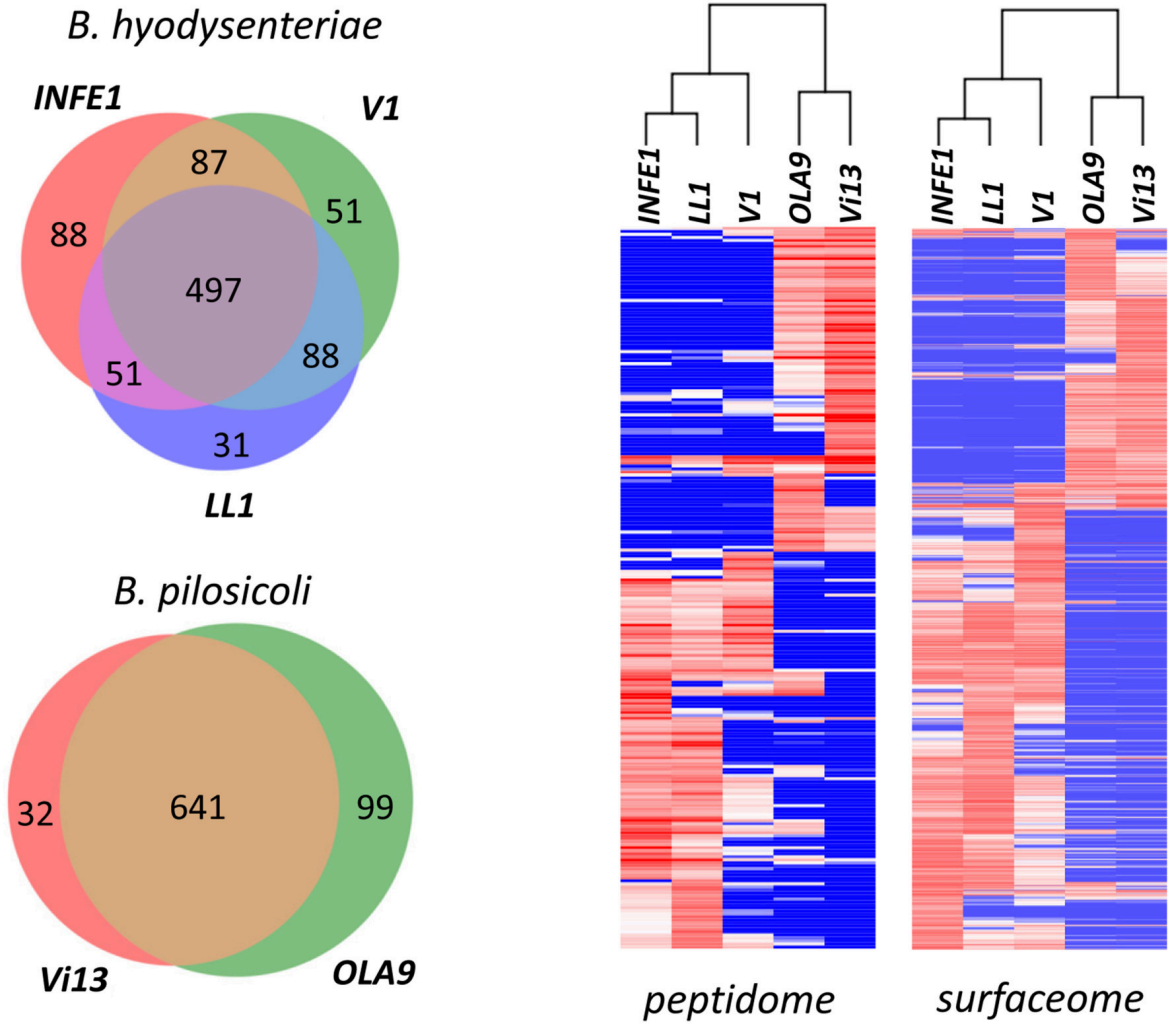
**Comparison of the ***Brachyspira*** strains**. **(Left)** Number of proteins identified in the different *Brachyspira* strains analyzed. Only proteins with more than 15 PeptideShaker-validated spectra were considered. **(Right)** Hierarchical clustering of the peptide sequences identified in the different fractions from each *Brachyspira* strain. Only sequences showing a standard deviation >10 for the PSM observed in the different strains were considered for clustering. The cluster images are highly compressed because they include 240 and 853 peptides for the peptidome and surfaceome, respectively. Full-size cluster figures, including peptide sequence clusters, are shown in Figure S5.

The different strains analyzed produced similar numbers of protein identifications, with a high degree of overlap between collections (Figure 8, Left). Among *B. hyodysenteriae* strains, however, more differences were found than among *B. pilosicoli* strains (an average of 16% of strain-exclusive accessions for any pair of *B. hyodysenteriae* strains compared with 8% for *B. pilosicoli*). These differences increase when only surfaceome-exclusive proteins are considered. In this case, an average of only 42 of 307 surfaceome proteins were common to any pair of *B. hyodysenteriae* strains (Table S9), whereas on average 140 of 385 surfaceome proteins were common to the two *B. pilosicoli* strains (Table S10). This is in contrast to observations made at the genomic level in a study in which the genomes of 20 strains of *B. hyodysenteriae* were sequenced, aligned, and compared (Black et al., 2015). As a reference for *B. pilosicoli*, the study used the genome of four *B. pilosicoli* strains described by Mappley et al. (2012). The comparison was also extended to the gene protein prediction level; this indicated that protein homology between strains ranged from 75.6 to 88.5% for *B. hyodysenteriae* and from 54.9 to 68.4% for *B. pilosicoli*. Based on the high homology between strains of *B. hyodysenteriae* observed in that study, the authors concluded that the genome of this species is clonal whereas that of *B. pilosicoli* is recombinant. The disagreement with our findings could be due to the small number of strains considered in our study, to the fact that our collection of proteins represents a subset of the proteome (exposed proteome) or to the difference between predicted gene products and their actual expression levels. Proteomic analysis of a larger number of strains would be necessary to decide among these possibilities.

Cluster analysis of the abundance data (indirectly measured as the number of PSM) of the identified peptide sequences shows a clear distinction between the two species (Figure 8, Right). Using either the surfaceome or the peptidome data, *B. pilosicoli* and *B. hyodysenteriae* strains cluster in two different branches of the dendrograms. In the case of the *B. hyodysenteriae* strains, the *V1* strain presents differences with respect to the *LL1* and *INFE1* that place these strains in two different sub-branches.

#### Genes related to virulence

By analogy with secreted and surface proteins of other pathogenic organisms, secreted proteins and proteins expressed on the surface of *B. hyodysenteriae* and *B. pilosicoli* can play an important role in colonization and disease expression (Trott et al., 2001). Bellgard et al. screened 314 protein coding sequences potentially involved in pathogenesis and virulence in the *B. hyodysenteriae* WA1 genome (Bellgard et al., 2009). The potential virulence genes included genes coding for proteins involved in lipopolysaccharide biosynthesis, adhesion, chemotaxis, and motility, host cell membrane degradation, nutrition, immunoevasion, and immunosuppression. The same group also described 235 genes that are potentially involved in pathogenesis and virulence in *B. pilosicoli* 95/1000 (Wanchanthuek et al., 2010). Many of the products of these virulence-related genes were found in the fractions analyzed in our study (examples are given in Table S11).

The large number of genes involved in chemotaxis and motility in these *Brachyspira* species reflects the importance of these functions in relation to their enteric lifestyle and the colonization process (Bellgard et al., 2009). To induce disease, the highly motile spirochetes colonize colonic crypts and enter goblet cells, from which they induce a characteristic outpouring of mucus (Bellgard et al., 2009; Hampson and Ahmed, 2009). The capacity for movement in this dense environment is one determinant of the bacterium's virulence, and some non-chemotactic strains have been demonstrated to be avirulent (Milner and Sellwood, 1994). The chemotaxis-related protein assignations with higher numbers of validated peptide matches in our collection are methyl-accepting chemotaxis proteins B (McpB) that are present mainly in the surfaceomes of both species (Table 3). Other methyl-accepting proteins detected included several accessions from McpA and McpC proteins that were observed only in *B. hyodysenteriae*. The absence of the *mcpC* gene in *B. pilosicoli* strains was previously noted by other authors, whereas *mcpA* has been detected in both species (Wanchanthuek et al., 2010; Mappley et al., 2012). The most highly represented chemosensory transducer gene product was the chemotaxis protein CheY, which is found in the surfaceome, and exoproteome fractions of the two species studied. Other highly expressed proteins in this class were the chemotaxis histidine kinase CheA and the chemotaxis response regulator protein-glutamate methylesterase CheB. Less abundant chemotaxis proteins identified were, in decreasing order of PSM, CheW, CheX, CheD, CheR, and CheC. Unlike the other members of the family, CheW showed higher counts (ca. 3:1) in the exoproteome than in the surfaceome fractions.

Previous studies have described as many as 33 (Wanchanthuek et al., 2010) and 42 (Mappley et al., 2012) genes related to flagella in *B. hyodysenteriae* and *B. pilosicoli*, respectively (Hampson and Ahmed, 2009). In current *Brachyspira* databases, a total of 52 different flagella-related gene names are annotated (42 and 40 for *B. hyodysenteriae* and *B. pilosicoli*, respectively), and a few others are annotated as ORF names. Here, we present evidence for 49 protein accessions covering 29 different genes related to the production and function of these organelles (including the *fla, flh, flg, fli*, and *mot* families). The abundance of flagellar proteins was found to differ between the two species, with ca. 4905 validated PSM in *B. hyodysenteriae* vs. 2577 PSM in *B. pilosicoli*. This could be related to the different numbers of flagella in these cells (*B. hyodysenteriae* is reported to have approximately 7–14 flagella/cell and *B. pilosicoli* to have 4–7 endoflagella; Hampson, 2012).

Several membrane proteins of *B. hyodysenteriae* have been described as mediators of adherence to host cells (Gömmel et al., 2013). This group includes Vsps, lipoproteins such as BmpB and SmpA and SmpB, and other integral/inner membrane proteins (Wanchanthuek et al., 2010).

Vsp proteins are considered the major protein component of the outer membrane of *B. hyodysenteriae*, and it has been shown that sera from affected pigs are immunoreactive toward these proteins (Witchell et al., 2011). Vsp proteins form stable multimeric complexes that, in *B. hyodysenteriae*, have been described as consisting mainly of VpsF but that can contain also VpsE, VpsI, and VpsD. We have identified 6 products of the genes *vspD, vspE, vspF*, and *vspH*, all of them found either preferentially or exclusively in the exoproteome fraction. The most-represented members are Q9AEX5 (VpsH) and F1CJQ5 (VpsF) from *B. hyodysenteriae* and D8ICU0 (VpsD) from *B. pilosicoli*. VspD was among the most abundant exoproteome-exclusive proteins identified in our analyses (Table 5); it was detected only in *B. pilosicoli* samples (Uniprot accession numbers D8ICU0 and J9UQJ0). Interestingly, we also detected this protein in western blot analyses in which protein extracts from two *B. pilosicoli* strains were incubated with sera from infected pigs; however, it was not detected when the same experiment was performed with *B. hyodysenteriae* strain *V1* (Rodriguez-Asiain, in preparation). The *vspD* gene has been described as a virulence factor in *B. hyodysenteriae* and was recently included in a list of 33 potential targets for the development of a vaccine against *B. hyodysenteriae* (Bellgard et al., 2015). Up to four different VspD main accessions have been annotated for *B. hyodysenteriae* (C0R1D9, F1CJQ3, COR1D9, and C0R1E2). Sequences O68157 and F1CJQ3 include one or several identical sequences annotated with different accessions. Whereas C0R1E2 is a short polypeptide, the other three sequences are proteins >40 KDa in size, a size similar to that of *B. pilosicoli* VspD proteins, and show more than 90% identity among them and with other surface proteins, such as VspC (F1CJQ2, O68156, and other identical sequences) and VspB. No peptide from any of these sequences was identified in any *B. hyodysenteriae* fraction despite the fact that, except for C0R1E2, these proteins are expected to be as detectable as the analogous proteins of *B. pilosicoli*. These results suggest that VspD may be expressed in very low amounts in *B. hyodysenteriae*. A search of VspD peptides in extracts from the full proteomes of *B. pilosicoli* (ATCC #51139, strain P43/6/78) and *B. hyodysenteriae* (ATCC #49526, strain WA-1) confirmed these results (Casas et al., in preparation). Whereas up to 10 peptides from VspD could be found in the *B. pilosicoli* extract, only one was detected in the *B. hyodysenteriae* sample. The most abundant *B. pilosicoli* VspD peptide was found with more than 100 validated PSM, whereas the only *B. hyodysenteriae* VspD peptide was found with 4 PSM. These figures suggest a difference in expression of approximately 25-fold.

Several membrane lipoproteins and membrane proteins involved in lipoprotein biosynthesis were identified. The detected lipoproteins included five BmpB sequences that were present in the exoproteome and surfaceome fractions; the most abundant were C0R281 (*B. hyodysenteriae*) and J9UFV3 (*B. pilosicoli*). Two accessions for the outer membrane proteins SmpA and SmpB were also detected in the *B. hyodysenteriae* samples and in the surfaceome fraction. SmpA, a membrane-associated lipoprotein localized in the outer surface of the spirochete, has been proposed as a vaccine candidate (Holden et al., 2006; Hidalgo et al., 2010). The genes *smpA* and *smpB* are related, although different. Individual strains of *B. hyodysenteriae* contain either *smpA* or *smpB* but not both. In a study of ca. 40 isolates from Spanish pigs, all presented the *smpA* gene (Hidalgo et al., 2010), whereas the two genes were equally represented in a small group of Australian, UK, and North American strains (Holden et al., 2006). In our study, strain *INFE1* expressed the *smpB* product (seven validated peptides, 47% protein sequence coverage), whereas strain *LL1* expressed SmpA (four validated peptides, 30 % protein sequence coverage). These results agree with the PCR characterization of the strains (Table 1). However, for strain *V1*, whose PCR characterization indicated the presence of the *smpA* gene sequence, neither SmpA nor SmpB could be identified in the proteomics analysis. A search of the PeptideShaker unfiltered data allowed us to detect two non-validated assignations, each with just one spectral match, to SmpB peptides in *V1*. The matches corresponded to very low-quality spectra, so our results may indicate low, or no expression of any of these two proteins in *V1*.

Many other membrane proteins were identified in our collection, including members of the carbohydrate phosphotransferase system (PTS), the most abundant of which was C0QW75, a transmembrane protein of *B. hyodysenteriae*. These proteins were mainly identified in the surfaceome, and several members of this class (Ptsg and FruA) were among the more abundant surfaceome-specific proteins (Table 4). Phosphoenolpyruvate PTS components have recently been described as modulators of virulence in *Borrelia burgdorferi* (Khajanchi et al., 2015).

Hemolysins are considered major virulence factors that may be involved in the disruption and shedding of colonic enterocytes, a process that exposes the underlying lamina propria to polymicrobial invasion, and inflammation. Up to 8 and 12 genes have been predicted to encode hemolysin in *B. hyodysenteriae* and *B. pilosicoli*, respectively (Wanchanthuek et al., 2010; Mappley et al., 2012; Black et al., 2015). We identified seven hemolysin proteins from the *hly* and *tly* genes, the most abundant of which was C0R0R9 (*hlyB, B. hyodysenteriae*) and, with slightly fewer validated spectra (18 vs. 65), D8IFI0 (*B. pilosicoli*). Except for C0R0R9, which was also present in the exoproteome fraction, the hemolysins were preferentially located in the surfaceome.

Another important set of virulence factors are those related to aerotolerance. Although *Brachyspiras* are anaerobic bacteria, they support aerobic conditions inside their hosts (i.e., oxygen in mucosal tissues; Stanton, 2006). The *nox* product, NADH oxidase, plays an important role in the metabolism of oxygen and the response to oxidative stress in these *Brachyspira* species. Mutant strains lacking *nox* genes were described as being 100- to 10,000-fold more sensitive to oxygen exposure than normal cells and were also shown to be less virulent (Stanton et al., 1999). Several highly abundant *nox*-derived proteins were identified both in the surfaceome and exoproteome fractions; NADH oxidase was among the 10 most abundant proteins detected in these fractions (Tables 2–3). In addition, several aerotolerance proteins (BatA, BatB, BatC, and BatE) were detected in much lower amounts.

Proteins related to iron uptake and metabolism are crucial for these bacteria, which live in an environment in which this essential element is stored intracellularly by the host. In Gram-negative bacteria, transport of free iron from the periplasmic space into the cytoplasm is proposed to occur by a classic ABC transporter system (Higgins, 1992). In *B. hyodysenteriae*, a transport system composed of three periplasmic iron-binding lipoproteins (BitA, BitB, and BitC), an ATP-binding protein (BitD) and two cytoplasmic membrane permeases (BitE and BitF) has been described (Dugourd et al., 1999). In our collection we detected 9 Bit proteins, the most frequent being C0QZS5 (BitD), C0QZS3 (BitB), C0QZS4 and O54369 (BitC), and C0QZS2 (BitA). These proteins were found in the exoproteome and surfaceome fractions, with a preference for the latter. We also detected one of the permeases, BitE (C0QZS6). This protein was found only in the surfaceome, although with a low number of validated matches.

Ankyrin-like proteins are delivered to the host cell by many bacterial pathogens. These proteins are known to bind to the host chromatin and to play a critical role in the interaction of the bacterial pathogen with the host cell (Alvarez-Ordóñez et al., 2013). Up to 55 ankyrin accessions from *B. hyodysenteriae* and *B. pilosicoli* were present in our collection. Those with the highest number of peptide matches (57% of total for ankyrins) were C0QW34 (BRAHW) and D8IDE2 (BRAPL). Ankyrins were primarily present in the exoproteome and surfaceome fractions (in ca. 2:1 proportion) and were practically absent in the peptidome data. Among the proteins with more than 10 validated PSM, five ankyrin accessions were characterized as exoproteome-exclusive, whereas five were exclusively found in the surfaceome. All the exoproteome-exclusive proteins with LipoP assignation (three proteins) had an SPI signal peptide, also predicted by SignalP, whereas all the surfaceome-exclusive proteins assigned by LipoP showed an SPIIase cleavage site or were assigned as transmembrane proteins.

Several genes with gene transfer agent (GTA) functionality have been detected in *Brachyspira* species (Motro et al., 2009). These elements permit the interchange of DNA fragments among cells and contribute to the genomic diversity of the species (Lang et al., 2012). The virus of *Serpulina hyodysenteriae* (VSH-1) genes are responsible for the first natural gene transfer mechanism described in spirochetes. This phage-like element consists of three modules encoding the capsid (head, seven genes), tail (seven genes), and proteins involved in cell lysis (seven genes); these genes are flanked by the bacterial genes *mcpB, mcpC, glt*, and *oxd* (Matson et al., 2005; Motro et al., 2009). Production of VSH-1 in *B. hyodysenteriae* is related to cell death and occurs after treatment of the cells with mitomycin C, H_2_O_2_, or antibiotics such as carbadox or metronidazole (Stanton et al., 2008; Lang et al., 2012). The VSH-1 structure contains random packages of DNA that can be incorporated into other cells and transfer antibiotic resistance or other virulence characteristics. It has been shown that *B. hyodysenteriae* strains acquire antibiotic resistance when exposed to VSH-1 particles from antibiotic-treated cells (Stanton et al., 2008). Several proteins from the VSH-1 capsid and tail were identified, including products of *hvp19, hvp28, hvp38*, and *hvp53*. In addition, our collection included several accessions with no gene assignation corresponding to sequences with 100% homology with products of *hvp13* and *hvp45*. The most abundant members were B9US97, B9US99, and B9US98 (*B. hyodysenteriae*) and B9US83 (*B. pilosicoli*), all of which were present mainly in the surfaceome and exoproteome fractions of the corresponding species (Table S5).

To gain more insight into the possible virulence factors present in our collection, we compared our data with the data available in the VFDB virulence factor database (http://www.mgc.ac.cn/VFs/; Chen et al., 2015). The VFDB (January 2016) describes nearly 26,400 virulence factors derived from 75 bacterial genera. For the analysis, we BLASTed our protein collection against VFDB, selecting the matches with higher BLAST scores (*e*-value < 0.01) and higher percentages of amino acid identities (>10%). The filtered collection (459 proteins) corresponded to virulence factors from 85 different bacterial species, with *Yersinia enterocolítica* the most frequent (Table S12). The most frequent virulence classes corresponded to flagellar proteins, followed by capsule-related proteins (mainly enzymes involved in carbohydrate biosynthesis), proteins of the Dot/lcm system (ankyrins), and oligopeptide-binding proteins. Many virulence factors corresponded to proteins related to sugar biosynthesis and metabolism. Among them were proteins related to galactose and mannose metabolism, the pentose phosphate pathway and peptidoglycan biosynthesis, all of which are involved in lipopolysaccharide (LPS) biosynthesis (Wanchanthuek et al., 2010). LPS are endotoxins situated in the outer membrane of Gram-negative bacteria. *Brachyspira* LPS differ from other Gram-negative species in that they contain the lipid A-sugar core of approximately 10–16 kDa but lack the repeating O-sugar ladder characteristic of these molecules. These lipooligosaccharides (LOS) are thought to be involved in the colonic damage produced by *B. hyodysenteriae. B. hyodysenteriae* LOS are known to be antigenic and are related to protective immunity against a specific serogroup (Wannemuehler et al., 1988). Among the genes involved in LOS biosynthesis, the more abundant products found were NagA (N-acetylglucosamine-6-phosphate deacetylase), NagB (glucosamine-6-phosphate deaminase), and the PTS N-acetylglucosamine-specific IIBC component, NagE. The *B. pilosicoli* deaminase D8IEE0 (NagB) was the most frequently detected (57% more validated PSM than the second most frequently detected protein); it was found exclusively in the surfaceome fraction, whereas the other *nag* products were detected both in the surfaceome and in the exoproteome. Other abundant proteins were products of the *murA, murC, glmU, lpxA, lpxM, kdsA*, and *rfbF* genes, but many other components of these biosynthetic pathways were also detected (see Table S11, LPS biosynthesis class).

## Conclusions

Considerable effort is being directed to the genome characterization of *Brachyspira* species and isolates; this is reflected in the rapid growth of the corresponding databases and the current availability of a reference proteome for *B. hyodysenteriae*. However, the number of genome products for which there is experimental evidence in these databases is still scarce, and no large-scale project has been directed to the study of the *Brachyspira* proteome. In this work, we present evidence for nearly 30,000 peptide sequences pertaining to more than 1000 proteins from different isolates of *Brachyspira*. This information constitutes a rich source of sequence data for proteogenomic studies. The large-scale characterization of peptides and proteins in the extracellular media as well as of exposed proteins on the bacterial surface provides evidence of the expression of proteins related to virulence factors associated with chemotaxis and motility, iron intake, aerotolerance, and LPS/LOS biosynthesis, and these proteins could be considered as candidates for the production of antibacterial vaccines. The quantitative information on the expression levels and location of these gene products supplied by the mass spectrometry analyses performed in this work gives further valuable information in this respect, as shown here for the reduced expression of vspD on the *B. hyodysenteriae* surface.

## Author contributions

JA, VC, and MC conceived the project and design the work. SV and CS supplied the biological samples and characterized the isolates by PCR. VC collected proteomics data. VC and JA analyzed and interpret the data. VC redacted manuscript draft and JA made the critical revision and produced the final manuscript. VC carried out this work in the framework of the Immunology Ph.D. program of the Autonomous University of Barcelona. All the authors approved the final version of the article.

## Funding

This work was funded by the Spanish MINECO (IPT-2011-0735-010000). The CSIC/UAB Proteomics Laboratory of IIBB-CSIC is a member of Proteored, PRB2-ISCIII and is supported by grant PT13/0001, of the PE I+D+i 2013-2016, funded by ISCIII and FEDER.

### Conflict of interest statement

The authors declare that the research was conducted in the absence of any commercial or financial relationships that could be construed as a potential conflict of interest.
