# Supplementary material for: The Exposed Proteomes of Brachyspira hyodysenteriae and B. pilosicoli
Source: Front Microbiol. 2016 Jul 21;7:1103. doi: 10.3389/fmicb.2016.01103 (PMC4955376; doi:10.3389/fmicb.2016.01103)
Supplement: Supplementary file 1 [file Presentation1.PDF]

SuppInfo

Content:

- Figure S1: Schematics of the samples and aliquots prepared and analyzed per experiment.

- Figure S2: PSORTb prediction for *Brachyspira* proteins.
  For the figure, proteins for which PSORTb predicts to have multiple locations, are classified as ***multiple***, independently of the predicted location of higher score. In the combined *B. hyodysenteriae* and *B. pilosicoli* databases, proteins with **multiple** location (4.8 % of the total) are distributed as **Unknown** (89%), **Cytoplasmic** (11%) and **Cytoplasmic membrane** (0.3%).
  Proteins with more than 5 PSMs were considered

- Figure S3: LipoP prediction for *Brachyspira* proteins.
  Proteins with more than 2 PSMs were considered.

- Figure S4: GOA annotation of the Molecular Function term for *Brachyspira* proteins.
  Proteins with more than 5 PSMs were considered

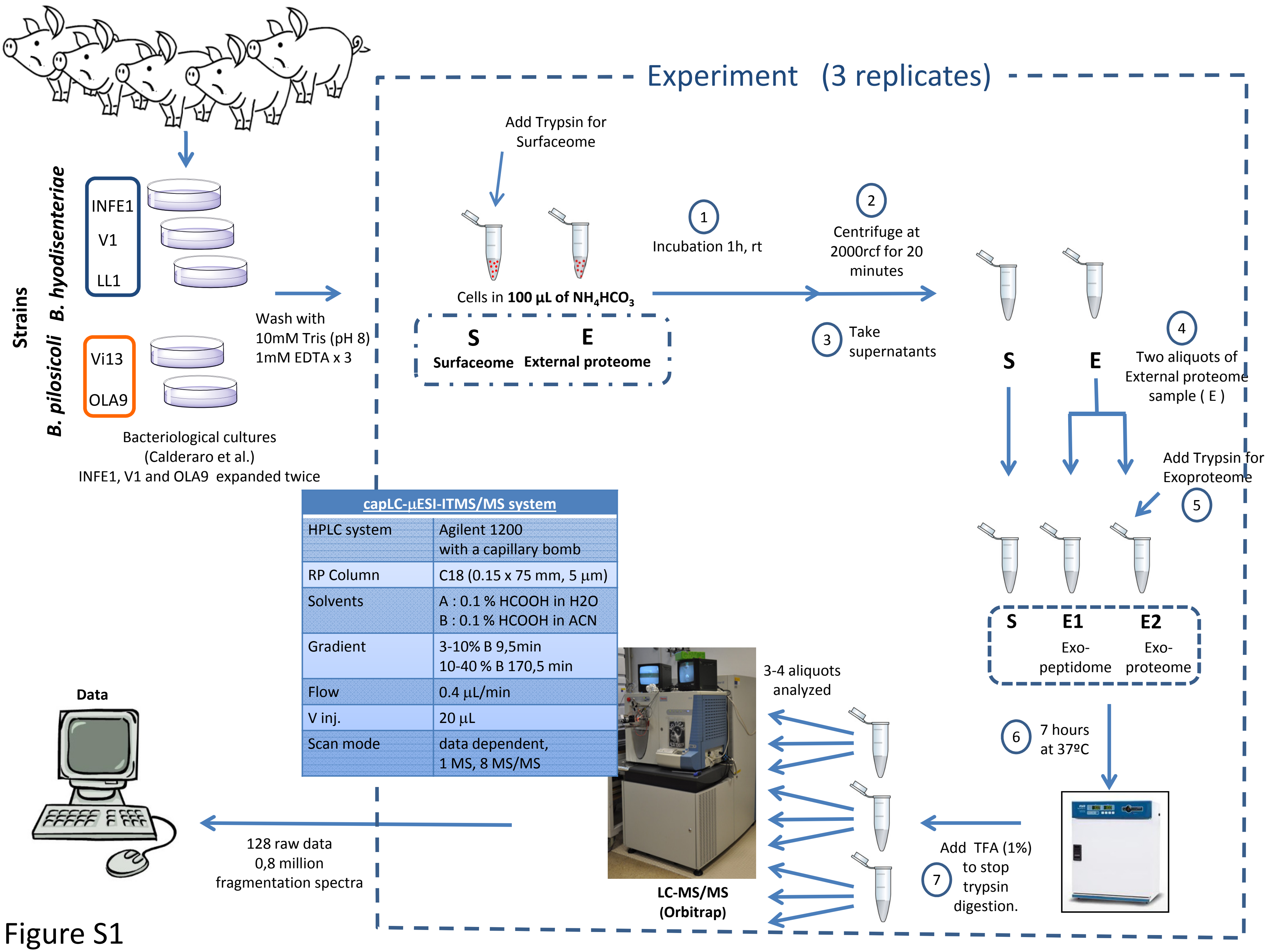

capLC-μESI-ITMS/MS system	
HPLC system	Agilent 1200 with a capillary bomb
RP Column	C18 (0.15 x 75 mm, 5 μm)
Solvents	A : 0.1 % HCOOH in H2O<br>B : 0.1 % HCOOH in ACN
Gradient	3-10% B 9,5min<br>10-40 % B 170,5 min
Flow	0.4 μL/min
V inj.	20 μL
Scan mode	data dependent,<br>1 MS, 8 MS/MS

Figure S1

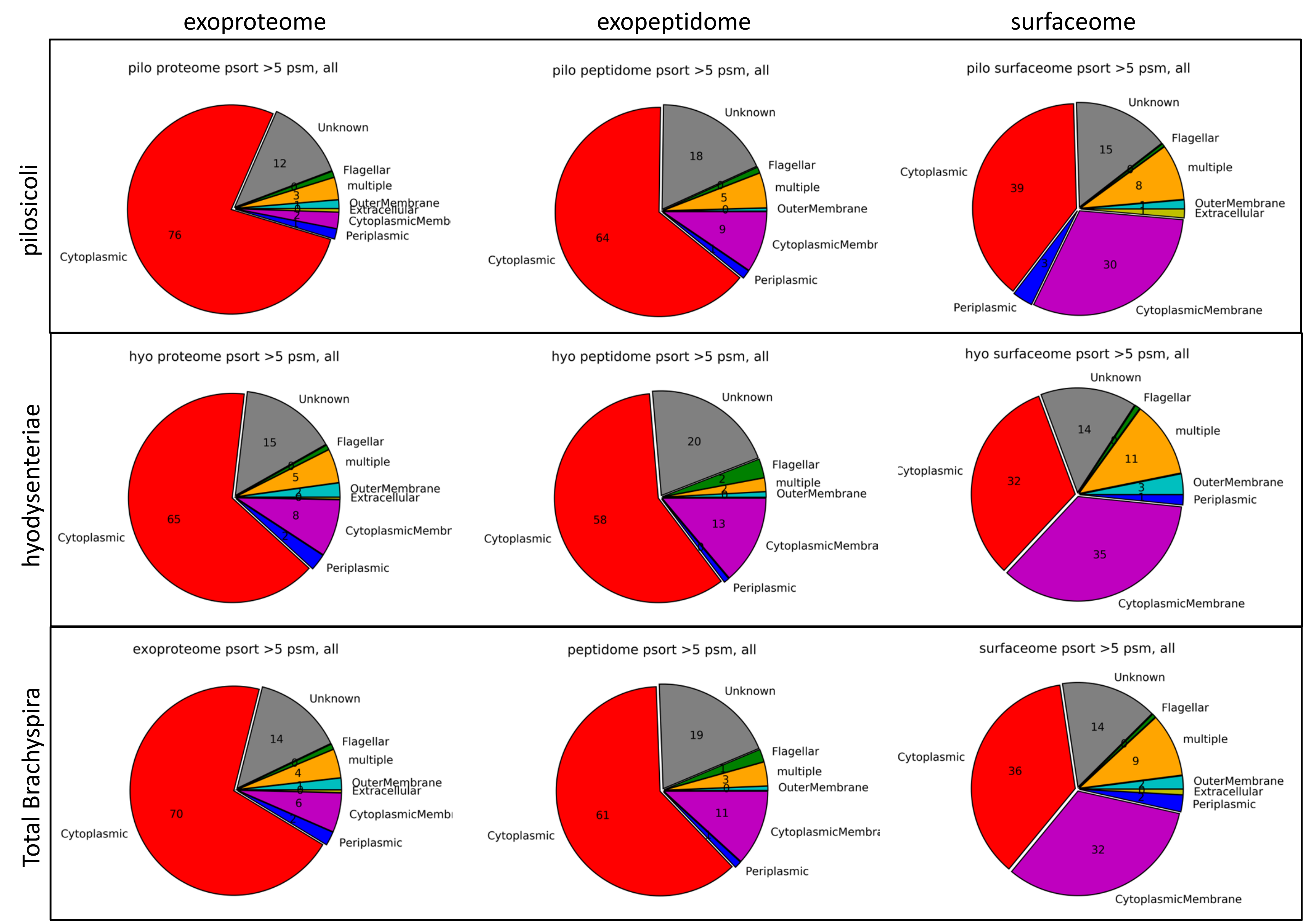
exoproteome
exopeptidome
surfaceome
pilosicoli
hyodysenteriae
Total Brachyspira
pilo proteome psort >5 psm, all
pilo peptidome psort >5 psm, all
pilo surfaceome psort >5 psm, all
hyo proteome psort >5 psm, all
hyo peptidome psort >5 psm, all
hyo surfaceome psort >5 psm, all
exoproteome psort >5 psm, all
peptidome psort >5 psm, all
surfaceome psort >5 psm, all
Unknown
Flagellar
multiple
OuterMembrane
Extracellular
CytoplasmicMembrane
Periplasmic
Cytoplasmic

Figure S2

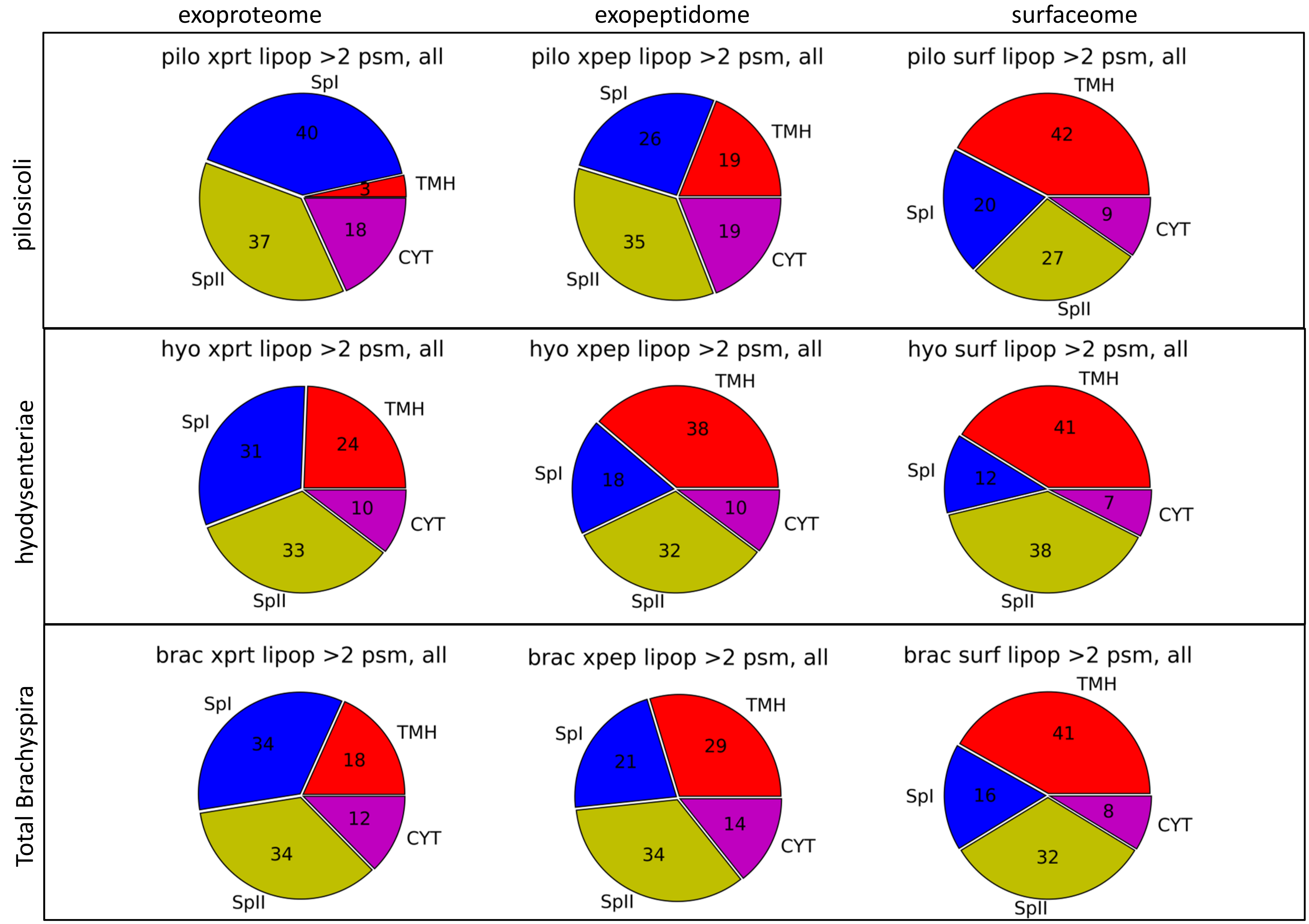
exoproteome
exopeptidome
surfaceome
pilosicoli
hyodysenteriae
Total Brachyspira
pilo xprt lipop >2 psm, all
SpI 40
TMH 3
CYT 18
SpII 37
pilo xpep lipop >2 psm, all
SpI 26
TMH 19
CYT 19
SpII 35
pilo surf lipop >2 psm, all
TMH 42
SpI 20
CYT 9
SpII 27
hyo xprt lipop >2 psm, all
SpI 31
TMH 24
CYT 10
SpII 33
hyo xpep lipop >2 psm, all
TMH 38
SpI 18
CYT 10
SpII 32
hyo surf lipop >2 psm, all
TMH 41
SpI 12
CYT 7
SpII 38
brac xprt lipop >2 psm, all
SpI 34
TMH 18
CYT 12
SpII 34
brac xpep lipop >2 psm, all
SpI 21
TMH 29
CYT 14
SpII 34
brac surf lipop >2 psm, all
TMH 41
SpI 16
CYT 8
SpII 32

Figure S3

GOA (STRAP) Molecular Function

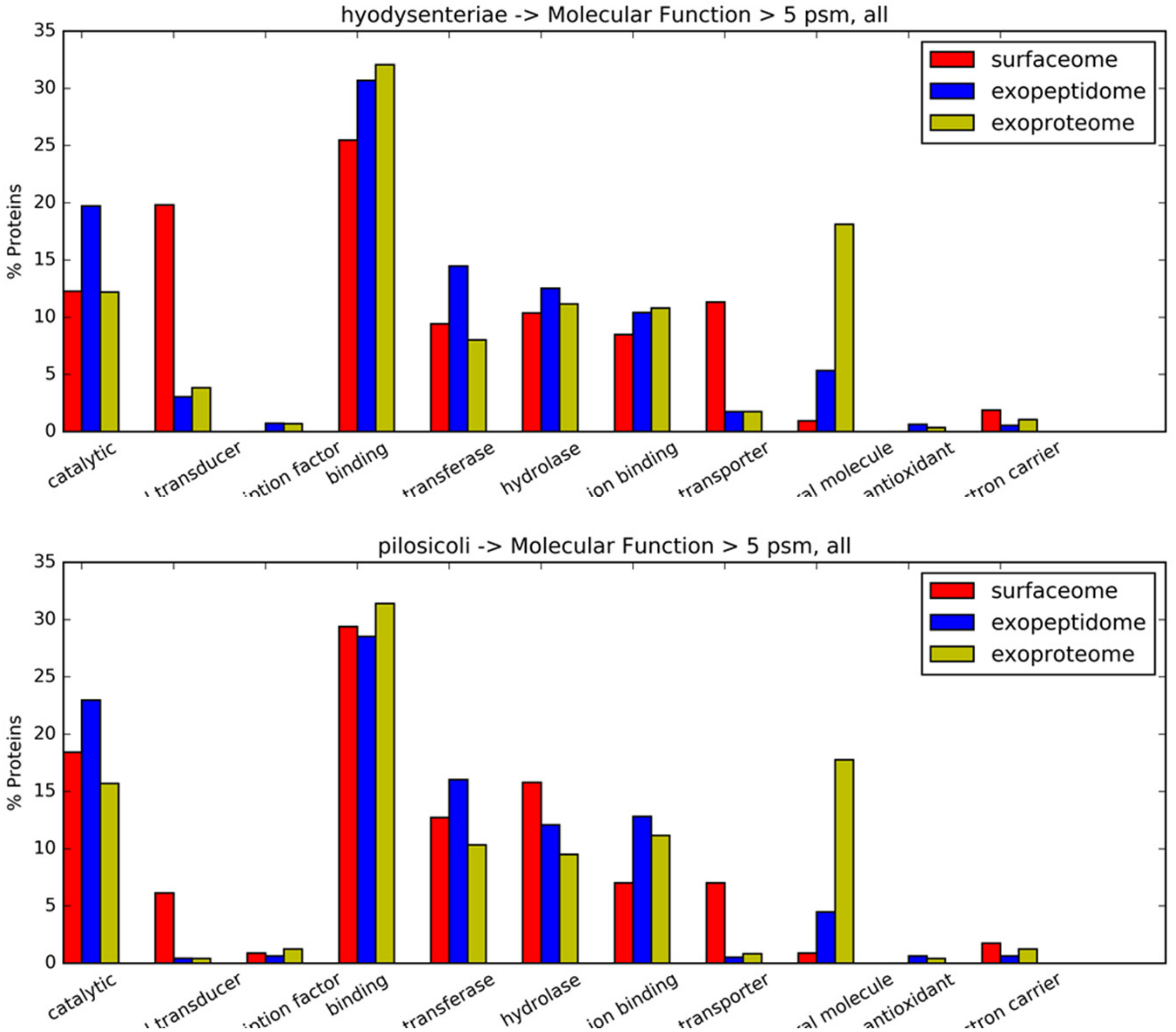

Figure S4
