# Supplementary material for: The Exposed Proteomes of Brachyspira hyodysenteriae and B. pilosicoli
Source: Front Microbiol. 2016 Jul 21;7:1103. doi: 10.3389/fmicb.2016.01103 (PMC4955376; doi:10.3389/fmicb.2016.01103)
Supplement: Supplementary file 2 [file Presentation2.pdf]

Figure S5

Full-size cluster figures

TOTAL COLLECTION

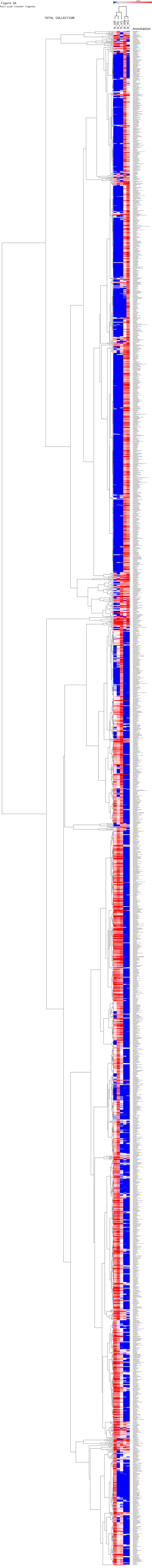

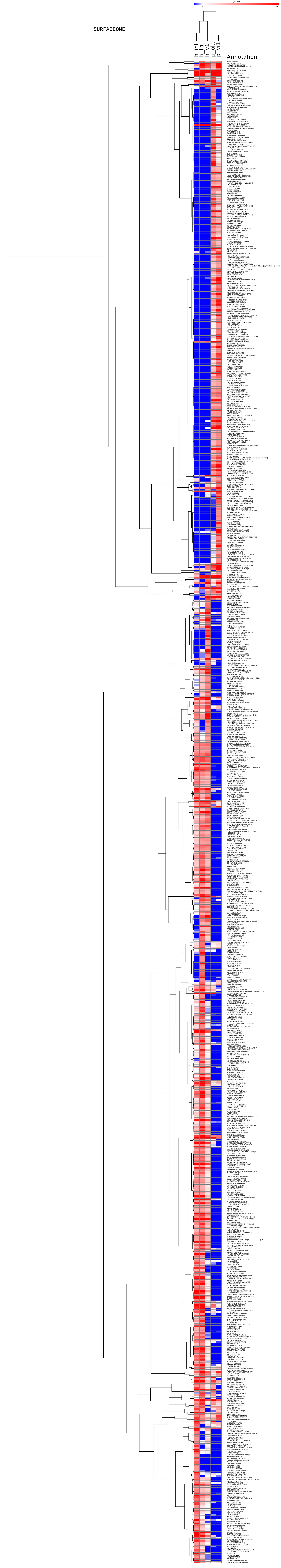
global
0
5
50
SURFACEOME
h_inf
h_ll1
h_v1
p_ola
p_vi1
Annotation

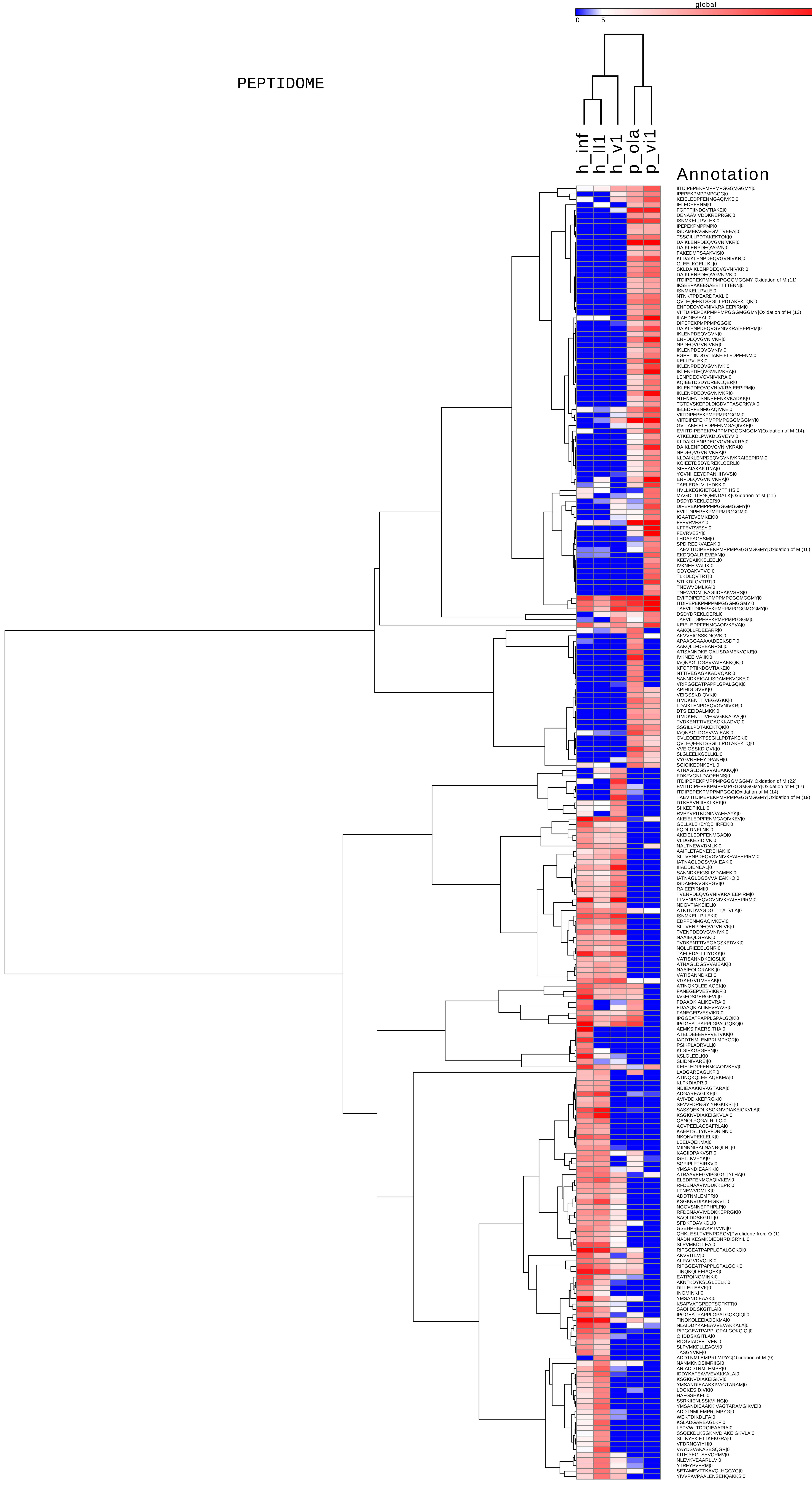

PEPTIDOME
global
0
5
h_inf
h_ll1
h_v1
p_ola
p_vi1
Annotation

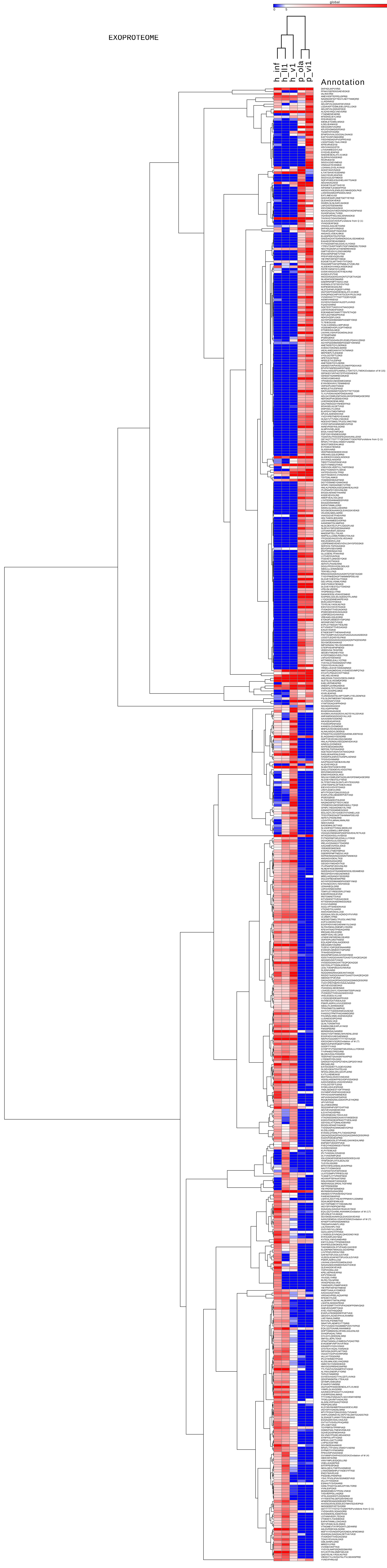

EXOPROTEOME
global
0
5
h_inf
h_ll1
h_v1
p_ola
p_vi1
Annotation
